# Physiology or Psychology: What Drives Human Emissions of Carbon Dioxide and Ammonia?

**DOI:** 10.1021/acs.est.3c07659

**Published:** 2024-01-18

**Authors:** Shen Yang, Gabriel Bekö, Pawel Wargocki, Meixia Zhang, Marouane Merizak, Athanasios Nenes, Jonathan Williams, Dusan Licina

**Affiliations:** † Human-Oriented Built Environment Lab, School of Architecture, Civil and Environmental Engineering, École Polytechnique Fédérale de Lausanne (EPFL), 1015 Lausanne, Switzerland; ‡ International Centre for Indoor Environment and Energy, Department of Environmental and Resource Engineering, Technical University of Denmark, Kongens Lyngby, 2800 Copenhagen, Denmark; § Laboratory of Atmospheric Processes and Their Impacts, School of Architecture, Civil & Environmental Engineering, École Polytechnique Fédérale de Lausanne (EPFL), 1015 Lausanne, Switzerland; ∥ Max Planck Institute for Chemistry, Hahn-Meitner Weg 1, 55128 Mainz, Germany; ⊥ Energy, Environment and Water Research Center, The Cyprus Institute, 2121 Nicosia, Cyprus

**Keywords:** skin temperature, electrodermal activity, heart rate, exercise, meditation, cognitive tasks

## Abstract

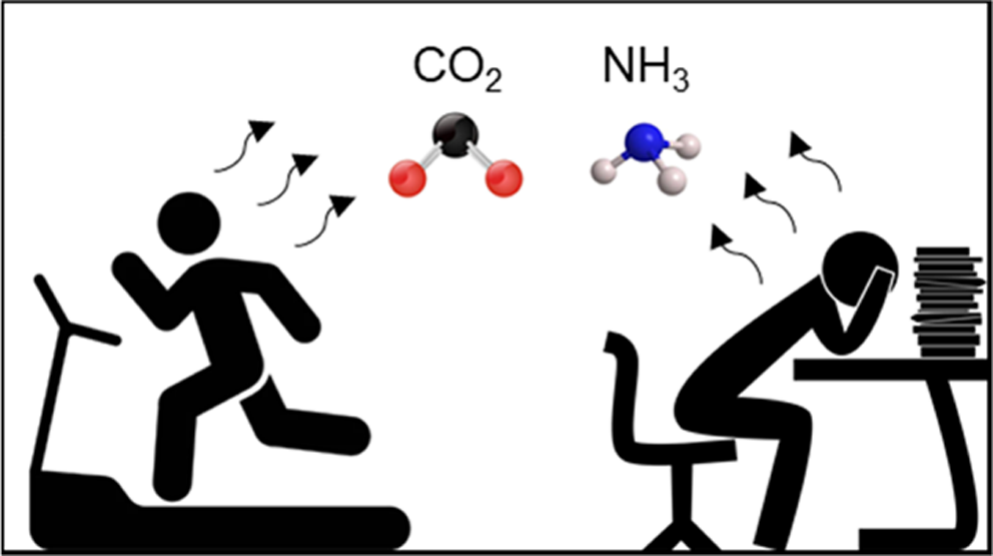

Humans are the primary sources of CO_2_ and NH_3_ indoors. Their emission rates may be influenced by human physiological and psychological status. This study investigated the impact of physiological and psychological engagements on the human emissions of CO_2_ and NH_3_. In a climate chamber, we measured CO_2_ and NH_3_ emissions from participants performing physical activities (walking and running at metabolic rates of 2.5 and 5 met, respectively) and psychological stimuli (meditation and cognitive tasks). Participants’ physiological responses were recorded, including the skin temperature, electrodermal activity (EDA), and heart rate, and then analyzed for their relationship with CO_2_ and NH_3_ emissions. The results showed that physiological engagement considerably elevated per-person CO_2_ emission rates from 19.6 (seated) to 46.9 (2.5 met) and 115.4 L/h (5 met) and NH_3_ emission rates from 2.7 to 5.1 and 8.3 mg/h, respectively. CO_2_ emissions reduced when participants stopped running, whereas NH_3_ emissions continued to increase owing to their distinct emission mechanisms. Psychological engagement did not significantly alter participants’ emissions of CO_2_ and NH_3_. Regression analysis revealed that CO_2_ emissions were predominantly correlated with heart rate, whereas NH_3_ emissions were mainly associated with skin temperature and EDA. These findings contribute to a deeper understanding of human metabolic emissions of CO_2_ and NH_3_.

## Abstract 2

This study reveals the influence of physiological and psychological activity on human emissions of CO_2_ and NH_3_ and their association with human physiological responses.

## Introduction

Carbon dioxide (CO_2_) and ammonia (NH_3_) are two important inorganic gases found in both outdoor and indoor environments. Outdoors, CO_2_, as a well-known greenhouse gas, strongly contributes to global warming and serves as a driver of climate change.^1,2^ NH_3_ is a major player in atmospheric chemistry, combining with acidic gases to form aerosol particles with direct implications for climate feedback and public health.^3,4^ Indoors, CO_2_ and NH_3_ tend to be the dominant acidic and basic species, respectively, governing the indoor acid–base balance and thus impacting indoor chemistry and air quality.^5−7^ For instance, NH_3_ has been found to be associated with nanocluster formation.^8^ The combination of CO_2_ and NH_3_ alters the pH of water films in indoor aerosols and on indoor surfaces and thus has meaningful impacts on the persistence of airborne viruses^9,10^ and indoor surface chemistry.^11^ Although in typical offices and residences, the CO_2_ and NH_3_ concentrations are insufficient to trigger acute health effects,^12,13^ elevated levels can still lead to discomfort and affect the performance of occupants.^14,15^

Humans are the major emitters of CO_2_ and NH_3_ in indoor environments. In addition to emissions from human activities such as cooking and cleaning,^16^ endogenous emissions from human metabolism contribute considerably to the buildup of indoor CO_2_ and NH_3_ levels. Humans emit CO_2_ mainly through exhalation; dermal emissions could account for only up to 3.5%.^17^ The emission rate depends on the metabolic rate, sex, age, and environmental parameters.^18,19^ Previous studies have reported per-person CO_2_ emission rates for adults ranging from 11 L/h during sleeping to ∼80 L/h during physical activities.^18−27^ Humans can also emit NH_3_ through exhalation.^28−34^ However, dermal emission is the dominating pathway of NH_3_ emission, an order of magnitude larger than emission from breath.^35−37^ NH_3_ emission rates range from 0.3 to 12 mg/h per person, depending on the age, clothing coverage, and air temperature.^35−37^

Physiological and psychological engagement are linked to human metabolic processes.^38,39^ The level of engagement can be reflected in several human physiological parameters such as skin temperature, electrodermal activity (EDA), and heart rate.^40−42^ Physiological engagement, such as doing physical exercise, elevates the emission rate of CO_2_.^23,24^ Performing cognitive tasks, a form of psychological engagement, has also been linked to higher CO_2_ emission rates, although the evidence is limited.^43^ Yet, the influence of physiological and psychological engagement and their relative importance for NH_3_ emissions from humans have not been well-documented. In addition, quantitative relationships between CO_2_ and NH_3_ emissions and human physiological responses are largely unknown.

In summary, although the relationship between physiological activities and human CO_2_ emissions has been well studied, there is a scarce body of research exploring the influence of psychological engagement on metabolic CO_2_ emissions from humans. Furthermore, there is a lack of research on the influence of physiological or psychological engagement on human NH_3_ emissions. The concurrent measurement of human CO_2_ and NH_3_ emissions also remains unexplored, which is important for elucidating their distinct emission behaviors and mechanisms. In addition, the correlation between the metabolic emissions of these two gases and human physiological responses is not well-understood. To contribute to existing knowledge, this study measured human metabolic emissions of CO_2_ and NH_3_ in a well-controlled climate chamber occupied by human subjects. We investigated the influence of physiological factors (walking and running) and psychological factors (meditation and cognitive tasks) on emission rates. We also recorded human physiological data, including skin temperature, EDA, and heart rate during the experiments and explored the relationships between CO_2_ and NH_3_ emissions and physiological responses. The results have the potential to deepen our understanding of emission behaviors and mechanisms of CO_2_ and NH_3_ from humans, enabling more accurate modeling of human emissions and their impacts on indoor air quality.

## Methods

### Climate Chamber

A climate chamber study has been demonstrated to be an effective approach for investigating human emissions of air pollutants.^8,35,44,45^ We performed a series of experiments in a 62 m^3^ climate-controlled chamber at the École Polytechnique Fédérale de Lausanne (EPFL) (Figure S1). The chamber was ventilated with 100% outdoor air that was filtered by a combination of a newly installed HEPA filter and an activated carbon molecular filter. The filtered air was distributed through a supply diffuser and exhausted via an outlet, both located in the ceiling. A dedicated Heating, Ventilation, and Air-Conditioning (HVAC) system controlled the air temperature and relative humidity inside the chamber at 24 ± 0.5 °C and 50 ± 5%, respectively. Two pedestal fans, located in the chamber corners and facing the walls, ensured efficient air mixing (Figure S1). Inside the chamber, three tablets were provided for the participants. In the experiments involving physiological engagement, the chamber was furnished with three treadmills, one table, and three chairs (Figure S1). The air change rate was controlled at 2.87 ± 0.04 h^–1^. In the psychological engagement experiments, there were three tables and four chairs inside the chamber (Figure S1). In these experiments, we adjusted the air change rate to 1.44 ± 0.01 h^–1^ to ensure adequate signals for the gas measurement instruments. The chamber and furniture surfaces were thoroughly cleaned prior to the experiments and at regular intervals during the campaign.

### Experimental Design and Procedure

We recruited four groups of three young adults in each group (age range: 19–32, BMI range: 21.5–29.3, see Table S1), labeled as G2A, G2B, G3A, and G3B. G2A and G2B were involved in the physiological engagement experiments, while the other two groups underwent psychological engagement. Each group consisted of two males and one female, except for G2B, which had one male and two females. The participants were asked to take a shower in the evening prior to the experiments, using the provided perfume- and odorant-free soap and shampoo. They were asked not to apply any personal care products. During the experiment days, the students were also asked to avoid drinking alcohol or eat spicy food, garlic, onions, and asparagus. On the experimental day, 30 min prior to entering the chamber, participants changed into short-sleeved T-shirts and shorts provided by the researchers. These new clothes were washed with perfume- and odorant-free laundry detergent immediately after purchase, tumble-dried, and sealed in individual zip-lock bags. In addition, all participants were provided with the same food and drink (a light sandwich with tomato and cheese and a bottle of water), which they finished consuming 10 min before entering the chamber. Participants were not allowed to bring any personal items into the chamber.

We performed physiological engagement experiments by asking participants to walk and run on treadmills. To investigate the influence of activity level on human emissions of CO_2_ and NH_3_, we asked participants to exercise at two metabolic rates: 2.5 and 5 met. Prior to the experiments, we gathered participants for a pretest session in order to adjust the treadmills to match the nominal metabolic rate of each participant (Table S1). Each experiment lasted for 2.5 h, including a 1 h preexercise sitting session, a 1 h exercise session, and a 30 min postexercise sitting session.

In the case of psychological experiments, participants engaged in two types of activities: online-guided meditation and cognitive tasks (d2 test^46,47^ and Stroop and multitasking test^48,49^). Each experiment consisted of two 45 min free-sitting periods with a 40 min engagement session in between. Details of the experimental design and procedure can be found in Section S1 and Table S2.

### Instrumentation and Quality Control

CO_2_ concentration inside the well-mixed chamber was measured by a high-accuracy gas analyzer at 0.5 s time intervals (LI-850, LI-COR Biosciences GmbH, Germany, range: 0–20,000 ppm, accuracy: ±1.5%). An air pump (AirCheck TOUCH, SKC Inc., U.K.) drew chamber air at 0.75 L/min into the gas analyzer. The NH_3_ level was monitored by a high-precision NH_3_ analyzer at 30 s time intervals (LSE NH_3_-1700 Analyzer, LSE Monitors, Netherlands, range: 0–15 ppm, noise: 1 ppb, precision: 2 ppb). The sampling flow rate was 140 mL/min. Both gas analyzers were placed outside the chamber, with sampling lines passing through a hole on the chamber wall at a height of 1 m. The NH_3_ sampling line was as short as possible (10 cm) to minimize NH_3_ loss in the sampling tube, whereas the length of the CO_2_ sampling line was ∼0.5 m. Inside the chamber, there were two air temperature and humidity sensors (HOBO U12–012, Onset Computer Co.) placed on the table.

In addition to measuring gases and environmental conditions, we equipped participants with wearable wristbands (E4, Empatica Inc.) to measure their physiological data. The wristbands recorded skin temperature (recording frequency: 4 Hz), electrodermal activity (EDA, 4 Hz), and heart rate (1 Hz). All participants were asked to put on wristbands on their left hands before entering the chamber.

All of the instruments were calibrated before the experimental campaign. As shown in Table S2, each experiment had one replicate, and the differences observed were generally within 15%, indicating good reproducibility of the experimental results.

### Data Analysis

We calculated per-person emission rates of CO_2_ and NH_3_ based on the material-balance equation, as described in Section S2. For statistical analysis and comparisons, the average emission rates were calculated for three subsessions of each experiment: before engagement, during engagement, and after engagement. The physiological data for each participant were averaged across the last 15 min of each subsession, as this period was found to approximate steady-state conditions of the activities. We performed paired *t* tests to examine the significance of the difference in emission rates and physiological data before, during, and after engagement. In addition, to investigate the relationship between gas emissions and human physiological data, we calculated 15 min averages of all parameters for the entire set of experiments. Subsequently, the 15 min average emission rates, physiological data, and chamber temperature and humidity during full experiments were analyzed by multilinear regression using MATLAB (2022b).

## Results and Discussion

### Characteristics of Gas Emissions and Physiological Data

**Figure 1:**
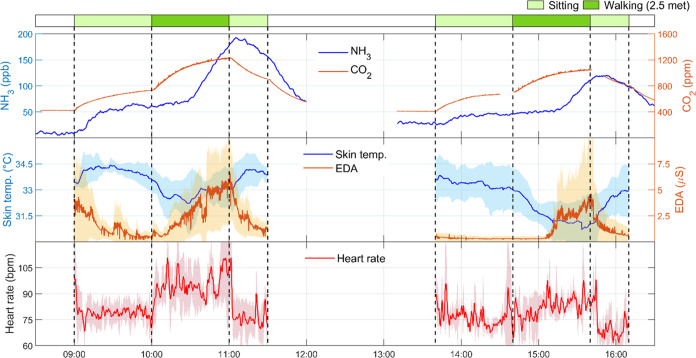
Time-series of NH_3_ and CO_2_ concentrations (top) and physiological data from participants: skin temperature and EDA (middle), and heart rate (bottom) in the experiments of physiological engagement by walking at 2.5 met. Morning and afternoon experiments were performed with participant groups G2A and G2B, respectively. The lines of the physiological data represent averages of all three participants in each group. Shaded areas represent standard deviation. Missing CO_2_ data represent periods of direct breath sampling (Section S1). Data are presented based on a single experimental day (date: August 19, 2022).

Figure 1 shows a time-series of NH_3_ and CO_2_ mixing ratios and human physiological data during the experiments of physiological engagement involving walking at 2.5 met. An instant increase of the CO_2_ and NH_3_ levels was observed after participants entered the chamber, indicating that humans are a potent source of these two gases. The physiological data gradually stabilized as CO_2_ and NH_3_ reached a steady state while participants remained seated. An immediate increase in CO_2_ levels occurred after the walking session began. However, the NH_3_ level started to climb up sharply only 30 min after participants began to walk. This difference demonstrates the distinct mechanisms underlying CO_2_ (respiratory) and NH_3_ (dermal) emissions.

The skin temperature initially dropped during walking for both groups and slightly recovered for group G2A, presumably owing to thermoregulation during moderate activities with an elevated metabolic rate and potential perspiration.^50−52^ Two mechanisms govern skin temperature variations during physiological exercise: peripheral vasoconstriction and vasodilation. The former contributes to the initial decrease in skin temperature while the latter increases the skin temperature. Both occur in response to changes in blood supply related to thermoregulation and increased metabolic heat production.^53,54^ During 2.5 met walking, peripheral vasoconstriction likely dominated. EDA and heart rate level increased, which is also consistent with previous findings.^42^ After the participants stopped exercising, the levels of CO_2_ inside the chamber decreased, whereas NH_3_ continued climbing for another 15 min and then gradually decreased. The skin temperature progressively increased, and EDA declined to prewalking levels. The heart rate rapidly returned to the value before walking, reflecting cardiovascular fitness.^55^ Relative to G2A, G2B exhibited a similar trend but lower CO_2_ and NH_3_ levels (see also Figures S2–S3 for the replicate experiments), probably owing to individual differences (average BMI of 26.6 and 21.5 kg/m^2^ in G2A and G2B, respectively).

**Figure 2:**
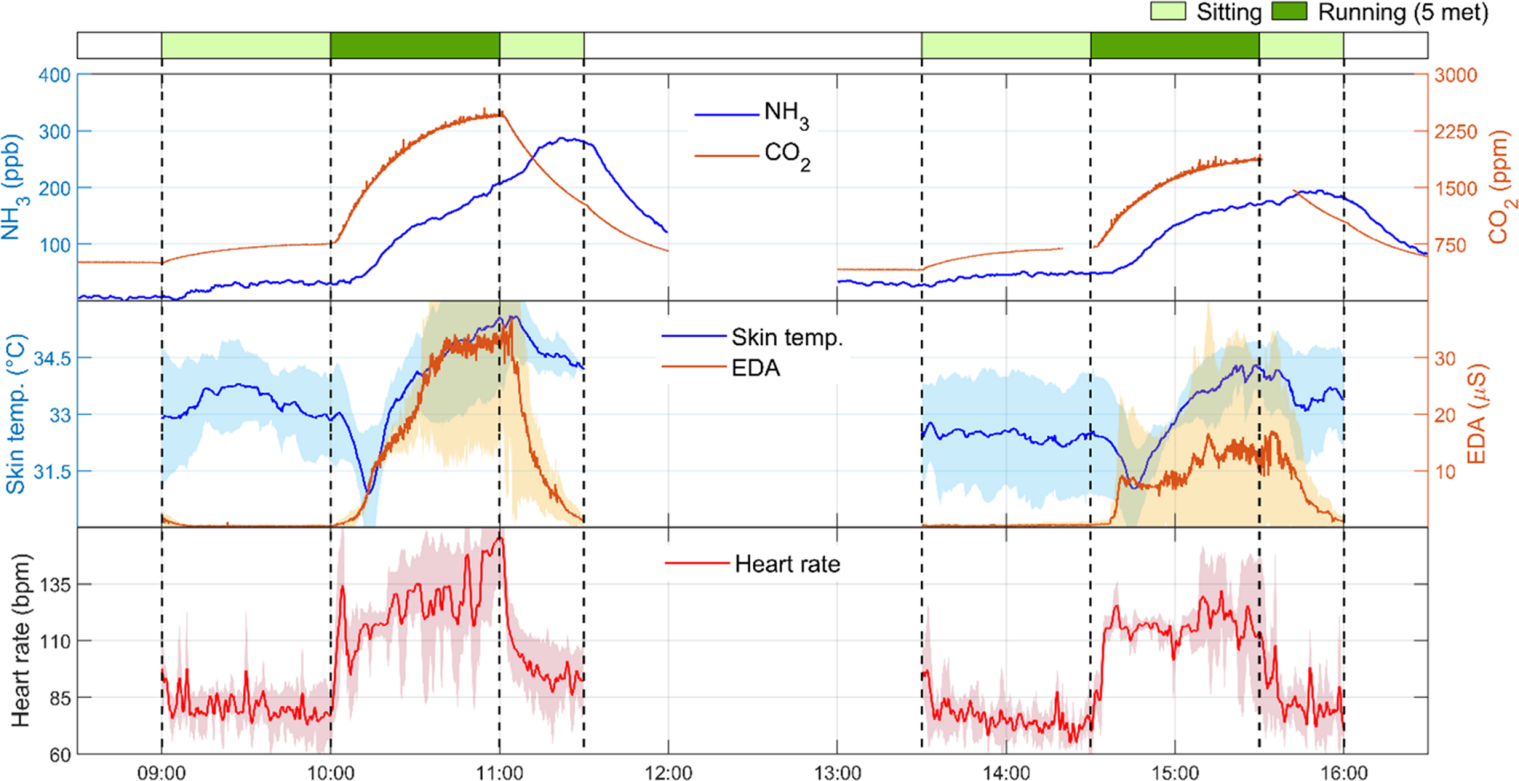
Time-series of NH_3_ and CO_2_ concentrations (top) and physiological data from participants: skin temperature and EDA (middle) and heart rate (bottom) in the experiments of physiological engagement by running at 5 met. Morning and afternoon experiments were performed with participant groups G2A and G2B, respectively. The lines of the physiological data represent averages of all three participants in each group. Shaded areas represent the standard deviation. Missing CO_2_ data represent periods of direct breath sampling (Section S1). Data are presented based on a single experimental day (date: August 23, 2022).

When the participants were physiologically engaged at a higher metabolic rate (5 met), the trends in indoor CO_2_ and NH_3_ levels, as well as human physiological data, were generally similar to those at 2.5 met but with considerably higher levels (Figure 2). After the participants stopped running, the NH_3_ level kept climbing and did not decrease until they exited the chamber. The skin temperature dropped rapidly at the beginning of running and then increased to higher level relative to prerunning. This indicates that the peripheral vasodilation tended to dominate during the 5 met running to cope with excess heat production.^47,48^ The skin temperature decreased slowly after the engagement stopped. The differences in the levels of CO_2_ and NH_3_, as well as physiological indicators remained pronounced between the two groups (see also Figures S4–S5).

**Figure 3:**
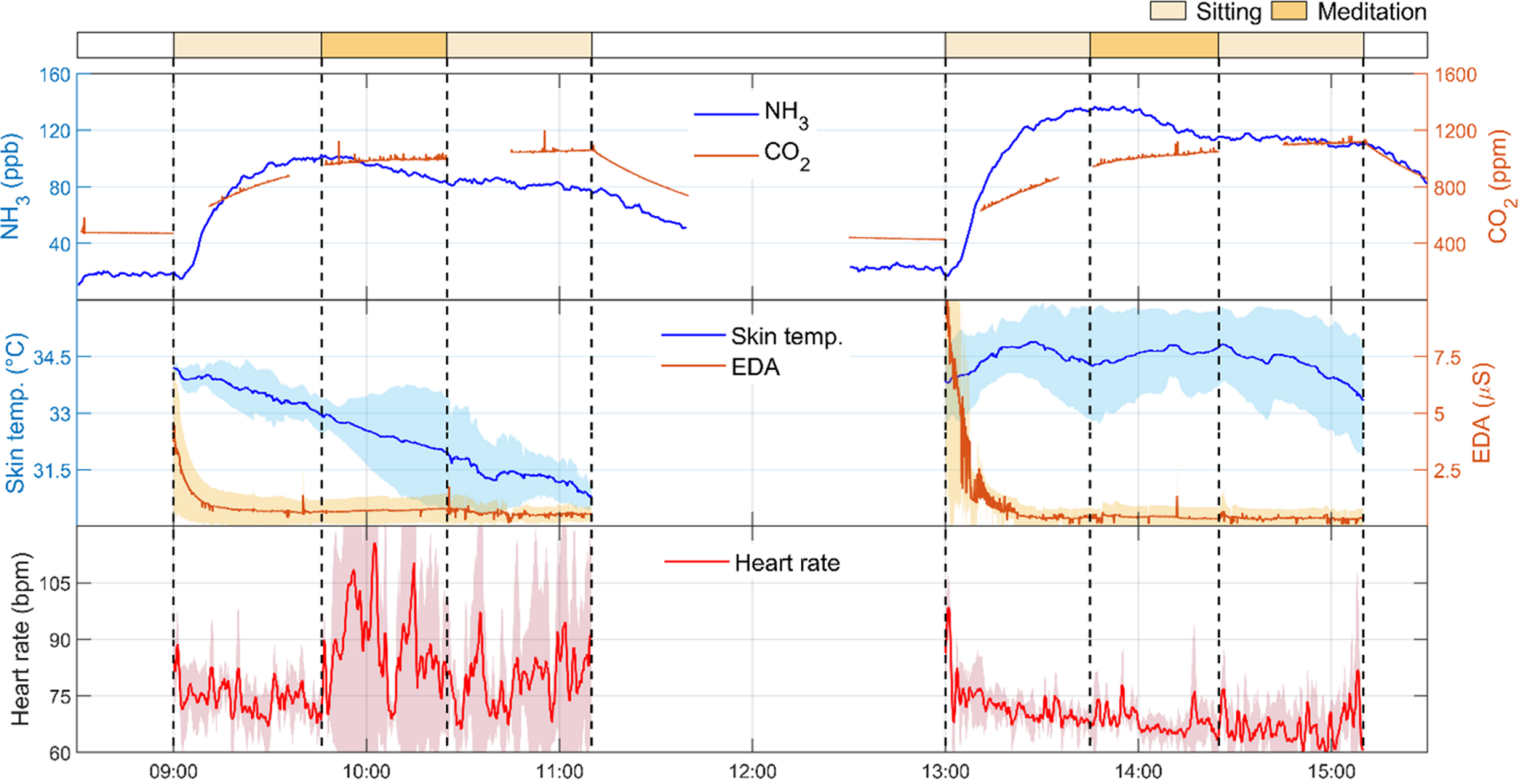
Time-series of NH_3_ and CO_2_ concentrations (top) and physiological data from participants: skin temperature and EDA (middle) and heart rate (bottom) in the experiments of psychological engagement by meditation. The morning and afternoon experiments were performed with participant groups G3A and G3B, respectively. The lines of the physiological data represent averages of all three participants in each group. Shaded areas represent standard deviation. Missing CO_2_ data represent periods of direct breath sampling (Section S1). Data are presented based on a single experimental day (date: August 26, 2022).

In meditation experiments, CO_2_ and NH_3_ concentrations did not vary as much as in the physiological engagement experiments, as shown in Figure 3 (see also Figures S6–S7). CO_2_ concentration increased and reached a steady state almost at the end of the experiment. NH_3_ exhibited a similar trend initially but slightly decreased after 1 h in the chamber, starting during the meditation period. The skin temperature generally followed a descending trend, especially for group G3A, in line with the fact that most participants felt slightly cool after settling down in the chamber (Figure S10), similar to previous findings.^56,57^ EDA levels reached a low value within 30 min after the participants entered the chamber and remained low during the rest of the experiment. The heart rate showed large variations, especially for group G3A, which was mainly caused by one participant, as made evident by the large standard deviation.

**Figure 4:**
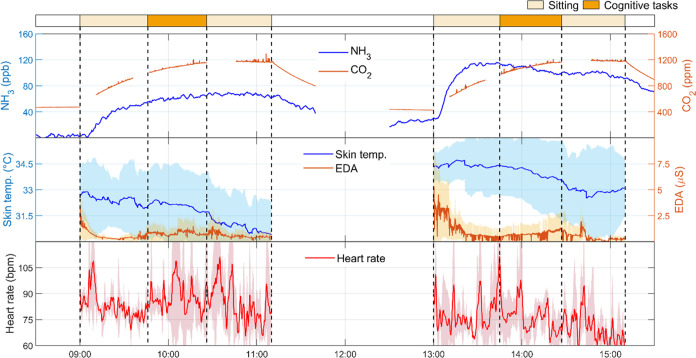
Time-series of NH_3_ and CO_2_ concentrations (top) and physiological data from participants: skin temperature and EDA (middle) and heart rate (bottom) in the experiments of psychological engagement by cognitive tasks. The morning and afternoon experiments were performed with participant groups G3A and G3B, respectively. The lines of the physiological data represent averages of all three participants in each group. Shaded areas represent standard deviation. Missing CO_2_ data represent periods of direct breath sampling (Section S1). Data are presented based on a single experimental day (date: August 31, 2022).

Cognitive tasks contributed to similar CO_2_ and NH_3_ concentrations as meditation, as illustrated in Figure 4 (see also Figures S8–S9). There were no meaningful changes in the CO_2_ and NH_3_ levels after the engagement started or ended. The skin temperature and heart rate also showed a descending trend and large variations, respectively. Notably, EDA levels exhibited a discernible difference: they slightly rose when the participants started cognitive tasks and fell back when the tasks ended. This echoes previous findings that cognitive stress induces changes in human EDA levels.^40^

### Emission Rate: Statistics and Comparisons

**Figure 5:**
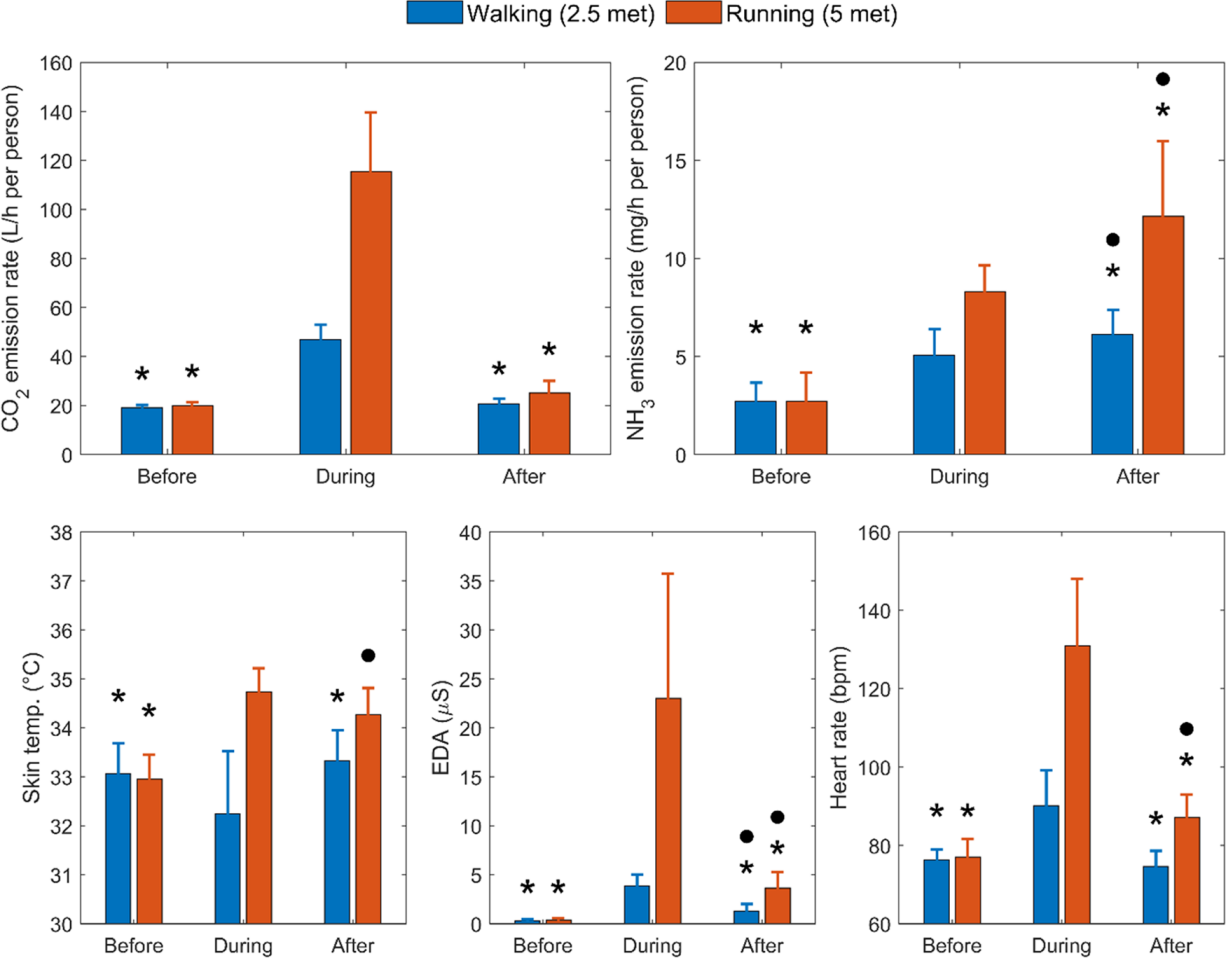
CO_2_ and NH_3_ emission rates (top) and human physiological data (bottom) before (1 h), during (1 h), and after (30 min) physiological engagement. The emission rates represent the average values across all respective subsessions (including replicates). The physiological data represent the average values of the last 15 min across all respective subsessions (including replicates), when they approximately reached steady state. The asterisks “*” indicate that the during–before or after–during difference was significant (*p* < 0.05). The dots “●” indicate that the after–before difference was significant (*p* < 0.05).

Figure 5 summarizes the average CO_2_ and NH_3_ emission rates and average steady-state human physiological data across all of the experiments with physiological engagement. The average CO_2_ emission rate when the participants were seated before exercise was 19.6 L/h per person, which is slightly higher than the average per-person CO_2_ generation rate in offices and conference rooms (17.3 L/h),^18^ probably owing to two participants with large BMI (29.3 and 25.6 kg/m^2^, respectively) in G2A (Table S1). The participants generated ∼2× and 5× more CO_2_ when walking at 2.5 met and running at 5 met, respectively. The increase was proportional to the designed metabolic rate. 30 min after walking, the CO_2_ emission rates returned to the prewalking level. Postrunning CO_2_ emission rates were slightly higher than the prerunning rates but without statistical significance (average: 25.2 vs 20.0 L/h, *p* = 0.09). The average per-person NH_3_ emission rate before running was 2.7 mg/h, within the range reported by Li et al. (0.57–5.2 mg/h for short-clothing scenarios).^35^ The NH_3_ emission rate increased ∼2× and 3× when participants exercised at 2.5 and 5 met, respectively. Unlike CO_2_, participants continued to emit NH_3_ at an elevated rate for 30 min after exercise. Regarding physiological data, the skin temperature significantly dropped during 2.5 met walking and returned to the previous level 30 min after the exercise session ended. In contrast, during 5 met running, the skin temperature significantly increased and remained high after running. Both EDA and heart rate sharply increased during exercise. 30 min after exercise, they decreased but remained significantly higher than the preexercise values, except for the heart rate after 2.5 met walking. However, it is foreseen that the EDA and heart rate would return to preexercise levels if participants had rested longer, given the steep decay of these two physiological signals after exercise stopped (Figures 1 and 2).

**Figure 6:**
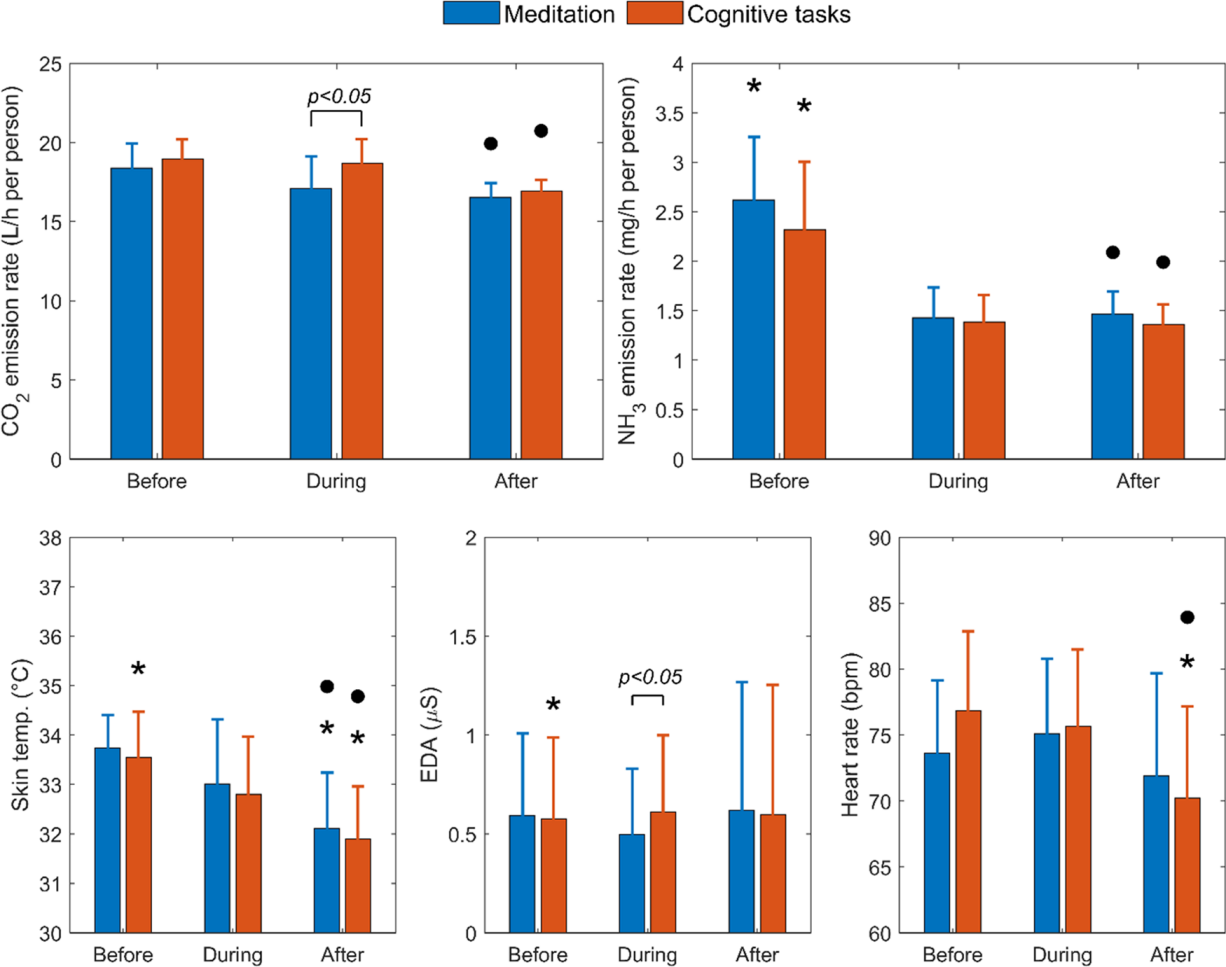
CO_2_ and NH_3_ emission rates (top) and human physiological data (bottom) before (45 min), during (40 min), and after (45 min) psychological engagement. The emission rates represent the average values across all respective subsessions (including replicates). The physiological data represent the average values of the last 15 min across all respective subsessions (including replicates) when they approximately reached steady state. The asterisks “*” indicate that the during–before or after–during difference was significant (*p* < 0.05). The dots “●” indicate that the after–before difference was significant (*p* < 0.05).

Both CO_2_ and NH_3_ emission rates in the psychological engagement experiments were within the range reported in the literature. We did not observe a significant change in CO_2_ generation during meditation or cognitive tasks relative to the preengagement session. However, when comparing between the two types of engagement, a slightly higher (*p* < 0.05) CO_2_ generation was found during cognitive tasks relative to meditation (Figure 6). The finding is in accordance with the study of Gall et al.^43^ that compared human CO_2_ emissions between relaxed and stressed activities. Nonetheless, it should be noted that their relaxed and stressed sessions were performed sequentially in a consecutive experiment, whereas this study investigated the two types of psychological engagement in separate experimental runs. A significant increase in EDA signals was found during cognitive tasks compared to before those tasks, as well as during meditation. NH_3_ emission rates decreased with time, following a similar trend with skin temperature reflecting the thermoregulation of the participants. Surprisingly, the heart rate did not exhibit a meaningful relationship with psychological engagement, except that after cognitive tasks, when the heart rate significantly decreased relative to during and before the engagement. To summarize, physiological engagement significantly elevated human emissions of CO_2_ and NH_3_, while the influence of psychological engagement was not significant.

The whole-body CO_2_ emission rates from sedentary humans have been widely reported in the literature. The average per-person CO_2_ emission rate during the preengagement sitting period across all experiments in this study was 19.0 ± 1.3 L/h (BMI: 24.1 ± 2.8 kg/m^2^). The value was somewhat higher than those from other chamber studies, such as 14.1 ± 3.3 L/h from Kuga et al.,^19^ 12.3 ± 1.7 L/h from Qi et al.,^22^ 16.8 ± 0.7 L/h from Gall et al.,^43^ and 16.1 ± 0.8 L/h from Sakamoto et al.^58^ and Li et al.^17^ In addition, Sakamoto et al. found higher CO_2_ emissions in the afternoon relative to the morning due to the increased metabolism from diet-induced thermogenesis.^58^ We did not observe such a difference, probably because we had two distinct groups of participants in the morning and afternoon sessions, and because the controlled diet and time of food consumption right before the experiments were the same in both sessions. Nevertheless, the CO_2_ emission rates in this study were within the range reported for sedentary adults (12.3–23 L/h per person).^18,22,59^ Moreover, the proportional increment of CO_2_ emission rate with increased metabolism (2.5 and 5 met exercise) echoes the linear relationship between the metabolic rate and the CO_2_ generation rate in the literature.^18^ Data on whole-body NH_3_ emission rate from humans are scarce at present. The average per-person NH_3_ emission rate during the preengagement sitting period across all of the experiments was 2.0 ± 0.9 mg/h, within the range reported by Li et al. for sedentary participants.^35^ To our knowledge, this study was the first to report human NH_3_ emissions during physiological and psychological engagements and to demonstrate that increased metabolism can elevate human emissions of NH_3_.

### Correlation and Regression

**Figure 7:**
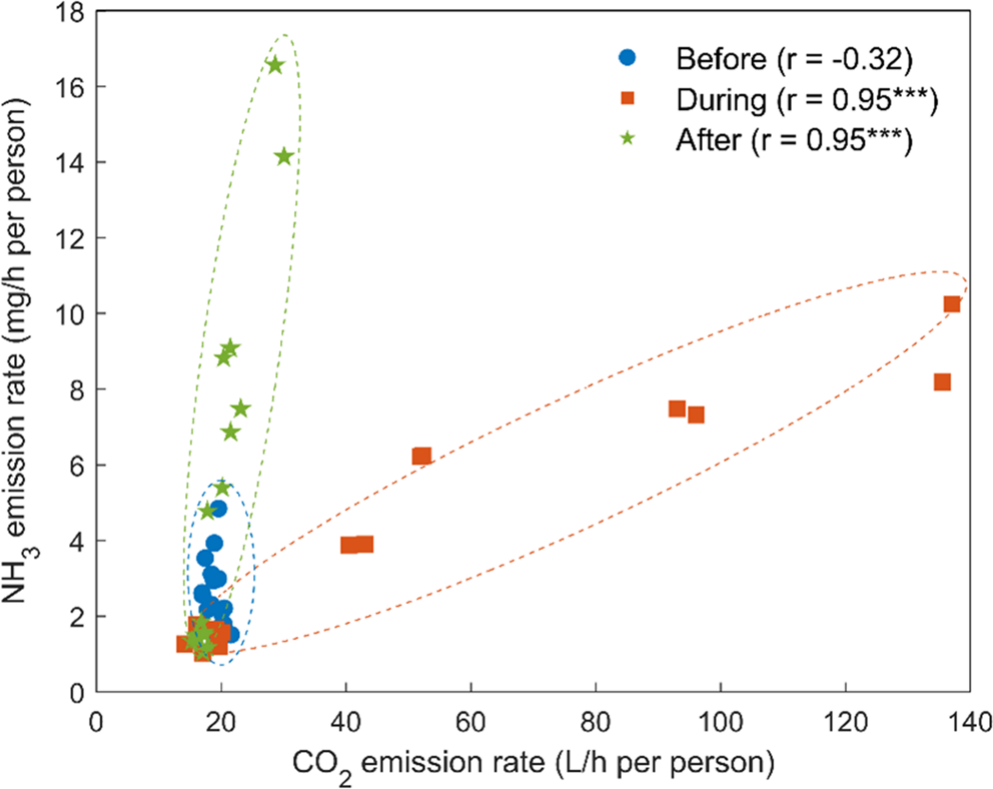
Pearson correlations between CO_2_ and NH_3_ emission rates before, during, and after physiological and psychological engagement. The data points include all emission rates obtained per subsession (48 in total). ***p < 0.001.

Previous findings in this study have demonstrated that CO_2_ and NH_3_ emissions are both related to human metabolic processes, although they have distinct emission mechanisms. Figure 7 shows the correlations between CO_2_ and NH_3_ emissions across all of the experiments. Even though the overall correlation was moderate (Pearson’s *r* = 0.48), when correlating the data separately during and after physiological and psychological engagement, we obtained strong and significant correlations between the emissions of the two gases. Such a correlation illustrates the “delayed” emission of NH_3_ relative to CO_2_ and reflects that the response to changes in human physiological status may take longer for dermal emissions relative to respiratory emissions. It may also be related to their difference in internal metabolism in human bodies. CO_2_ is a product generated from cellular respiration, after which it is transported in the bloodstream to the lungs and then expelled from the body via exhalation. In comparison, NH_3_ originates from protein metabolism and moves to the liver by the bloodstream, where it is converted to urea. NH_3_ remaining in the blood can diffuse through the skin or be emitted in sweat.^60^ The conversion of NH_3_ by the livers may take a longer time relative to the instant expulsion of CO_2_ by the lungs, leading to the “delayed” emission of NH_3_. In addition, NH_3_ excreted with sweating can be continuously emitted along with the evaporation of perspiration after exercise, which may also contribute to the “delayed” emissions. This may also be attributed to NH_3_’s stickiness, resulting in a longer equilibration time inside the chamber. A potential consequence of the lagged emissions is the elevated indoor base level after exercises that can alter indoor chemistry and pathogens.^9−11^

Regression analysis conducted across all of the experiments further highlights the differences between CO_2_ and NH_3_ emissions from humans, as seen in Table 1: 86% of the variability in CO_2_ emission rates could be explained by skin temperature, EDA, heart rate, air temperature, and relative humidity, with the heart rate being the most significant predictor, directly linked to the human metabolic rate. For NH_3_ emission rates, 58% of the variability could be explained by the same set of variables, with skin temperature and EDA potentially playing an important role (Table 1). Both of these are physiological indicators related to skin properties. However, given the lower *R*^2^ and the complexity of NH_3_ metabolism and emissions, the results should be interpreted with caution and a more detailed investigation of the biological processes affecting NH_3_ emissions is warranted. Although the selection of the data-averaging interval can influence the exact values of the coefficients (Table S3), the results remained similar when using average values for the whole periods of each subsession.

**Table 1:** Multilinear Regression Coefficients for CO_2_ and NH_3_ Emission Rates with Human Physiological Data (Skin Temperature, EDA, and Heart Rate) and Air Temperature and Relative Humidity^a^

	skin temperature	EDA	heart rate	air temperature	air relative humidity	intercept	adj. *R*^2^
CO_2_	–1.04 [*p* = 0.4]	0.60 [*p* = 0.1]	1.64*** [*p* = 10^–24^]	2.92 [*p* = 0.7]	–0.55 [*p* = 0.4]	–114.26	0.86
NH_3_	0.57** [*p* = 0.004]	0.25*** [*p* = 0.00003]	0.06* [*p* = 0.02]	–1.40 [*p* = 0.2]	0.02 [*p* = 0.6]	12.21	0.58

a The regression used 15 min of average data from all experiments (152 data points in total, both before, during, and after physiological and psychological engagement). *p < 0.05, **p < 0.01, and ***p < 0.001.

### Limitations

Several limitations should be acknowledged when interpreting the results. NH_3_ is known as a sticky gas that can be absorbed on chamber surfaces including walls, furniture, and human surfaces. This property may introduce bias to gas-phase NH_3_ measurements and emission rate calculation. The absorbed amount of NH_3_ onto surfaces depends on the gas-phase NH_3_ concentration (Section S3 and Figure S13), surface-bound NH_3_ (Section S3), surface properties, and air temperature and humidity.^61^ Given the case-specific NH_3_ absorption properties, obtaining a simple correction factor to adjust the measured NH_3_ or calculated emission rates is not feasible. We examined the uncertainty caused by the absorption/desorption processes of NH_3_, which was found to be within 13% (Section S3 and Table S4). In addition, NH_3_ emission rates reported in this study can be considered as a “net” rate, including both NH_3_ emission from and deposition onto humans.

Another potential source of bias to the emission rate calculation was the assumption of constant outdoor CO_2_ and NH_3_ levels during each experiment. Due to the limited number of instruments, we approximated the outdoor CO_2_ and NH_3_ levels using the 20 min average concentrations before participants entered the chamber and assumed them to be constant during the experiment. As we did not notice significant aperiodic variations of indoor CO_2_ and NH_3_ levels that were potentially caused by outdoor fluctuation, we expect that the assumption of a constant outdoor level during the experiments was justified.

Males and females have different chemical emission rates.^23^ This study, however, considered the average emissions in a mixed group and thus negated the potential influence of sex. In the experiments involving physiological engagement, we shortened the after-exercise subsession to 30 min considering the comfort of the participants after potential sweating. This period was insufficient for gaseous emissions and physiological data to return to preexercise levels, especially after 5 met running. In the experiments involving psychological engagement, the participants’ activities on the tablets were not strictly regulated before or after the engagement, which could bring some uncertainties in the during–before and after–during comparisons. Finally, air temperature and relative humidity were controlled within a narrow range in this study, which may have caused their insignificant predictive power in the linear regression models for CO_2_ and NH_3_ emissions (Table 1). Previous studies have demonstrated the influence of air temperature on CO_2_ and NH_3_ emissions.^26,35^ Hence, regression results presented in this study should be applied with caution.

### Implications and Future Outlook

Understanding the human emission rates of CO_2_ and NH_3_ is critical for indoor ventilation control and for ensuring acceptable indoor air quality for occupants. The findings of this study contribute to the knowledge of human emissions of air pollutants and the significance of human physiological and psychological factors, especially in the case of NH_3_, which has been relatively understudied. This knowledge lays the groundwork for constructing mathematical models for human-associated gas emissions that can be extrapolated to various indoor environments. These findings provide valuable insights into the dynamics of gas emissions and physiological responses during various engagement activities, shedding light on the complex interactions between human activities and indoor air quality.

In addition, in typical indoor environments with pronounced spatial gradients of air pollutants, CO_2_ and NH_3_ levels can be elevated in the perihuman microenvironment beyond those represented by the assumption of a well-mixed indoor environment, leading to elevated personal exposure.^62−66^ Future work exploring the effect of human gaseous emissions on personal exposure is warranted.

NH_3_ is the dominant neutralizer of acidity in indoor environments for airborne particles, aqueous surface films, and water bulk. Human emissions of NH_3_ are typically sufficient to neutralize the acidifying effects of exhaled CO_2_.^5^ However, given the strong influence of personal (e.g., clothing coverage) and environmental factors (e.g., air temperature) on human NH_3_ emissions^35^ and the inconsistent correlation between NH_3_ and CO_2_ emissions (Figure 7), future studies should consider broader emission scenarios to investigate the influence of human-emitted NH_3_ and CO_2_ on indoor acid–base balance and chemistry.

Human physiological data serve as indicators of a dynamic human–environment interaction. For instance, the skin temperature reflects the balance between human heat production and the thermal environment based on thermoregulation. The correlation between human gaseous emissions and physiological data reminds us of the importance of considering the interplay among the environment, human physiology, perception, and human emissions. Therefore, future studies focused on human emissions of air pollutants should not only consider environmental parameters and chemicals^16,67−70^ but also physiological indicators. Moreover, while this study has established preliminary correlations between gas emissions and human physiological indicators, the regression results should be interpreted with caution, given the complexity of metabolism and emission of the two gases. More detailed investigations into the biological, physical, and chemical processes associated with CO_2_ and NH_3_ emissions are warranted. In addition, the role of human perception of indoor environments (e.g., thermal and acoustics) in human emissions and consequent indoor air quality merits closer attention.

## Supporting Information Available

Details of experimental procedure (Section S1); calculation of CO_2_ and NH_3_ emission rates (Section S2); discussion about NH_3_ deposition onto chamber surfaces (Section S3); schematic layout of the climate chamber (Figure S1); time-series plot of measured parameters in the experiment of 2.5 met walking on August 19, 2022 (Figure S2); time-series plot of measured parameters in the experiment of 2.5 met walking on August 22, 2022 (replicate) (Figure S3); time-series plot of measured parameters in the experiment of 5 met running on August 23, 2022 (Figure S4); time-series plot of measured parameters in the experiment of 5 met running on August 24, 2022 (replicate) (Figure S5); time-series plot of measured parameters in the experiment of meditation on August 26, 2022 (Figure S6); time-series plot of measured parameters in the experiment of meditation on August 30, 2022 (replicate) (Figure S7); time-series plot of measured parameters in the experiment of cognitive tasks on August 29, 2022 (Figure S8); time-series plot of measured parameters in the experiment of cognitive tasks on August 31, 2022 (replicate) (Figure S9); thermal perception of participants collected 30 min after entering the chamber across all of the experiments (Figure S10); self-reported stress level from participants immediately before and after the 40 min psychological engagement session (Figure S11); d2 test sheet used in the psychological engagement experiments (Figure S12); difference between the NH_3_ removal rate and air change rate in relation to the average NH_3_ level during the occupied period in each experiment (Figure S13); physiological data of participants in each group and treadmill settings for each participant in the physiological engagement experiments (Table S1); summary of experimental conditions and associated average CO_2_ and NH_3_ emission rates, human physiological data, and chamber temperature and humidity (Table S2); multilinear regression coefficients for CO_2_ and NH_3_ emission rates with human physiological data and air temperature and relative humidity (Table S3); and NH_3_ removal rate in each experiment after participants left the chamber and comparison with air change rate (Table S4) (PDF)
