# Supplementary material for: Physiology or Psychology: What Drives Human Emissions of Carbon Dioxide and Ammonia?
Source: Environ Sci Technol. 2024 Jan 18;58(4):1986–97. doi: 10.1021/acs.est.3c07659 (PMC10832055; doi:10.1021/acs.est.3c07659)
Supplement: Supplementary file 1 — es3c07659_si_001.pdf [file es3c07659_si_001.pdf]

Supporting Information for

Physiology or psychology: What drives human emissions of carbon dioxide and ammonia?

Shen Yang,[1] Gabriel Bekö,[2] Pawel Wargocki,[2] Meixia Zhang,[1] Marouane Merizak,[1] Athanasios Nenes,[3] Jonathan Williams,[4,5] Dusan Licina[1]*

Affiliations

[1]Human-Oriented Built Environment Lab, School of Architecture, Civil and Environmental Engineering, École Polytechnique Fédérale de Lausanne (EPFL), 1015 Lausanne, Switzerland

[2]International Centre for Indoor Environment and Energy, Department of Environmental and Resource Engineering, Technical University of Denmark, Kongens Lyngby, 2800 Copenhagen, Denmark

[3]Laboratory of Atmospheric Processes and their Impacts, School of Architecture, Civil & Environmental Engineering, École Polytechnique Fédérale de Lausanne (EPFL), 1015 Lausanne, Switzerland

[4]Max Planck Institute for Chemistry, Hahn-Meitner Weg 1, 55128 Mainz, Germany

[5]Energy, Environment and Water Research Center, The Cyprus Institute, 2121 Nicosia, Cyprus

*Corresponding authors. Email: dusan.licina@epfl.ch

This file includes:

Section S1. Details of experimental procedure

Section S2. Calculation of CO_2 and NH_3 emission rates

Section S3. Discussion about NH_3 deposition onto chamber surfaces

Fig S1. Schematic layout of the climate chamber

Fig S2. Time-series plot of measured parameters in the experiment of 2.5-met walking on 19/08/2022

Fig S3. Time-series plot of measured parameters in the experiment of 2.5-met walking on 22/08/2022 (replicate)

Fig S4. Time-series plot of measured parameters in the experiment of 5-met running on 23/08/2022

Fig S5. Time-series plot of measured parameters in the experiment of 5-met running on 24/08/2022 (replicate)

Fig S6. Time-series plot of measured parameters in the experiment of meditation on 26/08/2022
Fig S7. Time-series plot of measured parameters in the experiment of meditation on 30/08/2022 (replicate)
Fig S8. Time-series plot of measured parameters in the experiment of cognitive tasks on 29/08/2022
Fig S9. Time-series plot of measured parameters in the experiment of cognitive tasks on 31/08/2022 (replicate)
Fig S10. Thermal perception of participants collected 30 min after entering the chamber across all the experiments
Fig S11. Self-reported stress level from participants immediately before and after the 40-min psychological engagement session
Fig S12. d2 Test sheet used in the psychological engagement experiments
Fig S13. The difference between NH_3 removal rate and air change rate in relation to average NH_3 level during occupied period in each experiment
Table S1. Physiological data of participants in each group and treadmill settings for each participant in the physiological engagement experiments
Table S2. Summary of experimental conditions and associated average CO_2 and NH_3 emission rates, human physiological data, and chamber temperature and humidity
Table S3. Multilinear regression coefficients for CO_2 and NH_3 emission rates with human physiological data and air temperature and relative humidity
Table S4. NH_3 removal rate in each experiment after participants left the chamber and comparison with air change rate

Supplementary Text

Section S1. Details of experimental procedure

Physiological engagement. Prior to the experiments, we gathered all the participants of G2A and G2B to pretest their metabolic rates at different treadmill settings, including running speed and inclination. We estimated the metabolic rate based on their individual age, gender, and weight, and measured heart rate during walking/running.[1] We obtained the treadmill setting for each participant to reach 2.5 and 5 met, respectively. A sticky note was placed on each treadmill to remind the participants of the treadmill settings for their designed metabolic rates.

On the experiment days, after entering the chamber, the group of three participants sat around the table for one hour, during which they were free to play with the provided tablets (Fig. S1). Afterwards, they were asked to walk/run on the treadmills at the adjusted speed and inclination according to the sticky note obtained from the pretest. The exercise session lasted for one hour. Then the participants stopped the treadmills and returned back to sit around the table for another 30 min before they exited the chamber. Each group participated in four half-day experiments, including two 2.5 met scenarios and two 5 met scenarios (replicate experiments). Hence, there were eight experiments with physiological engagement over four full days (Table S1).

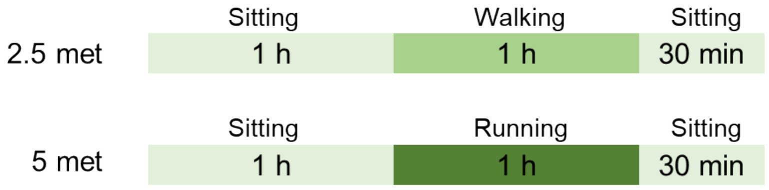

Psychological engagement. There were two types of psychological engagement in this study: online guided meditation and cognitive tasks. Groups of G3A and G3B participated in the experiments. To ensure a smooth understanding of the tasks by the participants, an instructor (female, age 31, BMI 23.1) accompanied the participants through the experiments when they were in the chamber. On the experiment days, the group of three participants and the instructor entered the chamber and sat at their tables for 45 min (Fig. S1). The participants were free to use the provided tablets.

For experiments with a meditation session, the participants followed the instruction from the instructor to put on the provided eye masks and earphones, and listen to a 40-min online meditation guide [2] without falling asleep.

For experiments with cognitive tasks, the participants were told before entering the chamber that the tasks were competitive and the best performer would receive prizes. This was done in order to arouse their engagement in the cognitive tasks. After the participants were seated for 45 min, the 40-min session included four consecutive tasks: 10-min d2 test [3,4] (Fig. S11, on papers), 10-min Stroop and multitasking test (on tablets),[5,6] 10-min d2 test, and 10-min Stroop and multitasking test. The instructor set an alarm clock to remind the participants to shift between the tests. After the 40-min session of meditation/cognitive tasks, the participants returned back to the tablets for another 45 min before exiting the chamber. Immediately before and after the 40-min engagement session, the participants were asked to fill a questionnaire about their subjective stress level at the specific moment (Fig. S12).

Each group participated in four half-day experiments, including two meditation scenarios and two cognitive-task scenarios (replicate experiments). Thus, there were eight experiments with psychological engagement over four full days (Table S1).

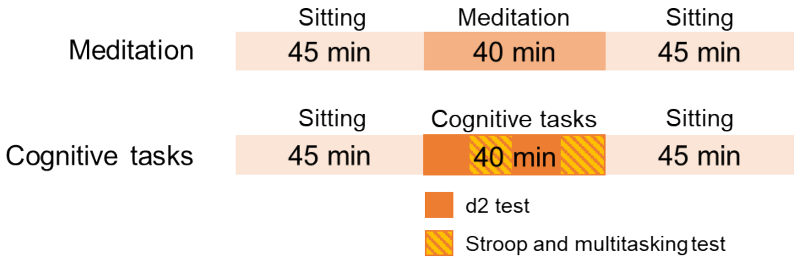

The chamber door was closed during the entire experiment. In each experiment, we asked the participants to fill a questionnaire on the tablets about their thermal perception 30 min after they entered the chamber. After they exited the chamber, the door was kept closed for 30 minutes to measure the decay of the gaseous compounds. For experiments in the morning, the chamber conditions were set the night before to ensure that they had reached steady state at the beginning of the experiment. After the decay period, the chamber was flushed with high air change rate (~ 9

h^{-1}) for 20 min to reduce the levels of CO_2 and NH_3 inside the chamber. Then the ventilation was set again to the designed air change rate (2.87 h^{-1} for physiological engagement and 1.44 h^{-1} for psychological engagement) to stabilize the chamber condition before the afternoon experiments.

It is noteworthy that we injected ozone into the chamber during all the 16 experiments to investigate ozone-human chemistry, which is not within the scope of this paper. The ozone concentration in the chamber was around 18-25 ppb, consistent with typical indoor and outdoor ozone levels. Such a level of ozone would not influence the CO_2 and NH_3 concentration inside the chamber.[7] Therefore, the factor of ozone injection was neglected in this study. In addition, in all the experiments, immediately before and after the engagement sessions, we asked one participant to move to the sampling station (Fig. S1) and stay for 10 min to breathe into a mouthpiece that was connected to the online monitor of chemical compounds. The breathing site was close to the CO_2 sampling line, and the CO_2 measurement may thus have been influenced by the breath sampling. The CO_2 data of these periods were excluded from the analysis, as shown in Fig. 1-4 and Fig. S2-S9.

Section S2. Calculation of CO_2 and NH_3 emission rates

The average emission rate of CO_2 and NH_3 during a given period was calculated based on the material-balance equation:

$$V\frac{\mathrm{d}C}{\mathrm{d}t} = nE - \alpha VC + \alpha VC_{out} \quad (1)$$

where V is the chamber volume, 62 m^3; C is the gas concentration measured inside the chamber; n is the number of occupants, 3 for physiological engagement experiments and 4 for psychological experiments; α is the air change rate, 2.87 h^{-1} for physiological engagement experiments and 1.44 h^{-1} for psychological experiments; C_{out} is the outdoor gas concentration, approximated by the 20-min average values before participants entering the chamber in each experiment; and E is the emission rate per person. Note that in psychological experiments, 3 participants and 1 instructor occupied the chamber. When calculating the average per-person emission rate, we assumed that the instructor experienced similar psychological conditions as the participants. The uncertainty caused by this can be considered minimal, given the insignificant influence of psychological engagement on CO_2 and NH_3 emissions (Table S2).

The solution of Equation (1) is:

$$C(t) = C_0 e^{-\alpha t} + (C_{out} + \frac{nE}{\alpha V})(1 - e^{-\alpha t}) \quad (2)$$

where C_0 is the initial gas concentration. Owing to the stability of CO_2, we performed a non-linear fitting (using MATLAB 2022b) of Equation 2 to obtain the per-person average CO_2 emission rate during a given period. As for NH_3, because of its sticky property, the concentration variation did not follow the trend of CO_2. Therefore, we approximated NH_3 emission rates by discretizing Equation 1, from which we got:

$$E = \frac{V}{n}(\alpha(\overline{C} - C_{out}) + \frac{C(T) - C(0)}{T})$$

(3)

where $\overline{C}$ is the average NH_3 concentration during a given period; $C(T)$ is the NH_3 concentration and the end of the given period (5-point average); $C(0)$ is the NH_3 concentration at the beginning of the given period (5-point average); and T is the duration of the given period. This discretization method has been widely used for emission rate calculation of "sticky" materials, such as airborne particles.[8,9]

<u>Section S3. Discussion about NH_3 deposition onto chamber surfaces</u>

As mentioned in Section S2, NH_3 is a sticky gas and thus has the potential to be absorbed onto the chamber surfaces, including chamber walls, furniture, and human surfaces. Such a sticky property of NH_3 may introduce bias to NH_3 emission rates calculated by Equation 3, as the emitted NH_3 may not fully remain in the gas phase for subsequent sampling by the NH_3 monitor. In order to estimate the uncertainty caused by surface absorption, we examined NH_3 removal rate during the decay period after participants exited the chamber in each experiment (listed in Table S4). The NH_3 removal rate is the sum of removal by ventilation and by surface absorption. Therefore, the difference between NH_3 removal rate and air change rate (ACH) represents the strength of surface absorption of NH_3 onto chamber surfaces.

As seen in Table S4, NH_3 removal rates in experiments with physiological engagement were generally lower than ACH (average: 2.58 vs. 2.87 h^{-1}). On the contrary, in experiments of psychological engagement, the NH_3 removal rates were mostly higher than ACH (average: 1.63

vs. 1.44 h^{-1}). This disparity can be partially explained by the difference in the average NH_3 level during occupied period in the experiments.

In physiological experiments, the NH_3 concentration inside the chamber was high and the chamber surfaces may have been saturated with NH_3. Therefore, after participants exited the chamber, NH_3 desorption from the surfaces overtook absorption, leading to lower removal rates relative to ACH. In contrast, in psychological experiments, the NH_3 levels inside the chamber were generally lower, which could be linked with NH_3 absorption on the surfaces. When the chamber became unoccupied, the absorption of NH_3 remained dominant over desorption, leading to higher NH_3 removal rates than ACH. In fact, we observed a negative correlation ($R^2 = 0.6$) when plotting the difference between NH_3 removal rate and ACH in relation to the average NH_3 level during occupied period in each experiment (Fig. S13), which supports the aforementioned presumption.

In addition to gas-phase NH_3 concentrations, NH_3 deposition onto surfaces can also be altered by surface-bounded NH_3, surface properties, air temperature, and humidity.[10] For instance, we observed that in most experimental days (6 out of 8), NH_3 removal rates were higher in the morning session relative to the afternoon (Table S4). This may be owing to the build-up of surface-bounded NH_3 level on chamber surfaces. Given the case-specific NH_3 absorption properties, obtaining a simple correction factor to account for NH_3 absorption on chamber surfaces when calculating the emission rates was not feasible. Nevertheless, the uncertainty in the obtained emission rates caused by such adsorption/desorption processes was generally within 13%, as shown in Table S4. The agreement in NH_3 emission rates between our results and the precedent study confirms the robustness of our experiments and calculations.[7]

It is also noteworthy that this uncertainty estimate only considers absorption to chamber surfaces including walls and furniture, but not human surfaces, such as human skin and clothing. In the occupied chamber, participants emitted NH_3, and the emitted NH_3 can deposit onto their skin and clothing surfaces. Such a deposition cannot be experimentally differentiated from human NH_3 emissions. Therefore, the emission rate reported in this study can be considered as a "net" emission rate, including both NH_3 emission from and deposition onto humans.

References

(1) Malchaire, J.; d'Ambrosio Alfano, F. R.; Palella, B. I. Evaluation of the Metabolic Rate Based on the Recording of the Heart Rate. *Ind Health* **2017**, *55* (3), 219–232. https://doi.org/10.2486/indhealth.2016-0177.

(2) *https://www.youtube.com/watch?v=jTJSJckA-oY&ab_channel=CarolineMcCreadyMeditation*.

(3) Bates, M. E.; Lemay, E. P. The D2 Test of Attention: Construct Validity and Extensions in Scoring Techniques. *Journal of the International Neuropsychological Society* **2004**, *10* (3), 392–400. https://doi.org/10.1017/S135561770410307X.

(4) Brard, M.; Lê, S. The Sequential Agglomerative Sorting Task, a New Methodology for the Sensory Characterization of Large Sets of Products. *J Sens Stud* **2019**, *34* (5). https://doi.org/10.1111/joss.12527.

(5) *https://www.psytoolkit.org/experiment-library/experiment_stroop.html*.

(6) *https://www.psytoolkit.org/experiment-library/experiment_multitasking.html*.

(7) Li, M.; Weschler, C. J.; Beko, G.; Wargocki, P.; Lucic, G.; Williams, J. Human Ammonia Emission Rates under Various Indoor Environmental Conditions. *Environ Sci Technol* **2020**, *54* (9), 5419–5428. https://doi.org/10.1021/acs.est.0c00094.

(8) Licina, D.; Tian, Y.; Nazaroff, W. W. Emission Rates and the Personal Cloud Effect Associated with Particle Release from the Perihuman Environment. *Indoor Air* **2017**, *27* (4), 791–802. https://doi.org/10.1111/ina.12365.

(9) Yang, S.; Beko, G.; Wargocki, P.; Williams, J.; Licina, D. Human Emissions of Size-Resolved Fluorescent Aerosol Particles: Influence of Personal and Environmental Factors. *Environ. Sci. Technol.* **2021**, *55* (1), 509–518.

(10) Li, J.; Xu, W.; You, B.; Sun, Y. Dynamic Variations of Ammonia in Various Life Spaces and Seasons and the Influences of Human Activities. *Build Environ* **2022**, *212*, 108820. https://doi.org/10.1016/j.buildenv.2022.108820.

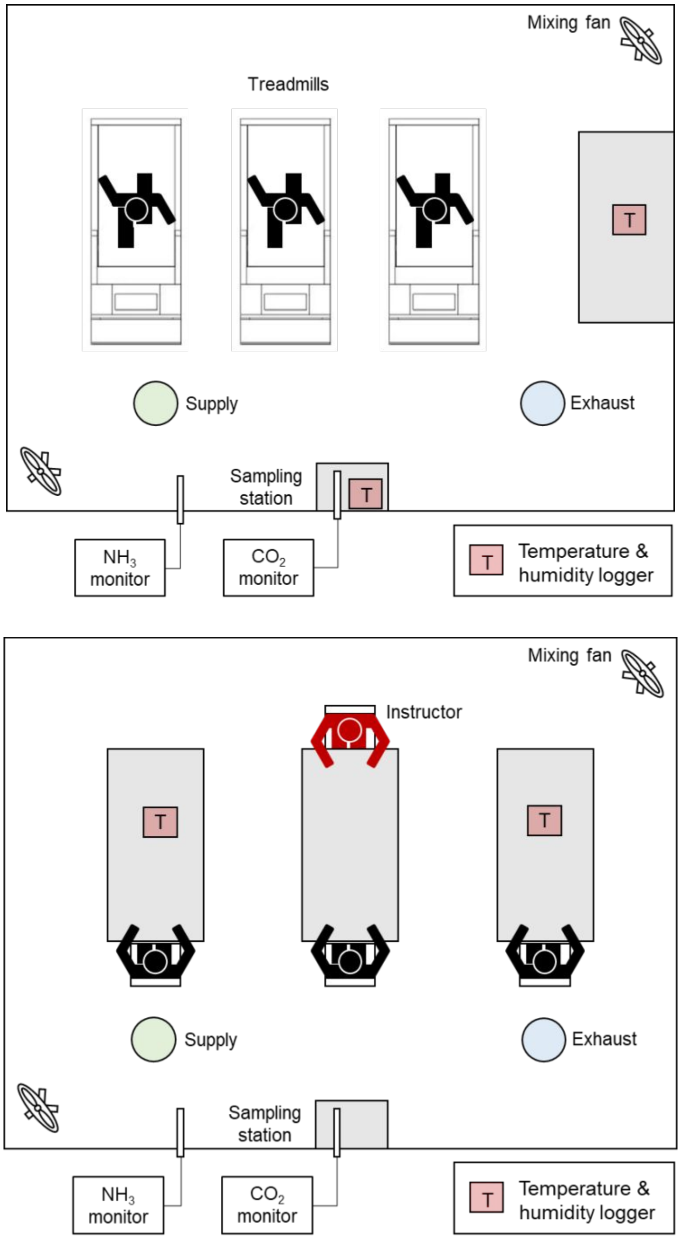

Fig. S1.

Schematic layout of the climate chamber with sampling locations for the experiments of physiological (upper) and psychological (lower) engagement. The air was supplied via the supply diffuser and exhausted through a single outlet in the ceiling. The supply air was 100% outdoor air after filtration by a combination of a newly installed HEPA filter and activated carbon molecular filters. The chamber wall was made of stainless steel, and the ceiling was covered by aluminum foils, whereas the floor was covered by vinyl plates. Two pedestal fans were placed at the corners facing the wall to ensure good mixing of the chamber air. Two-point CO_2 measurements (HOBO MX1102, Onset Inc., US) in the chamber at the places of temperature and humidity loggers showed <5% differences, indicating good air mixing. In the experiments of physiological engagement, three participants were running on the treadmills at speeds adjusted for each participant to achieve the specific metabolic rate. In psychological engagement experiments,

three participants were sitting at their own tables to perform either meditation or cognitive tasks. One additional person—an instructor—was seated in the chamber to guide the participants during the engagement.

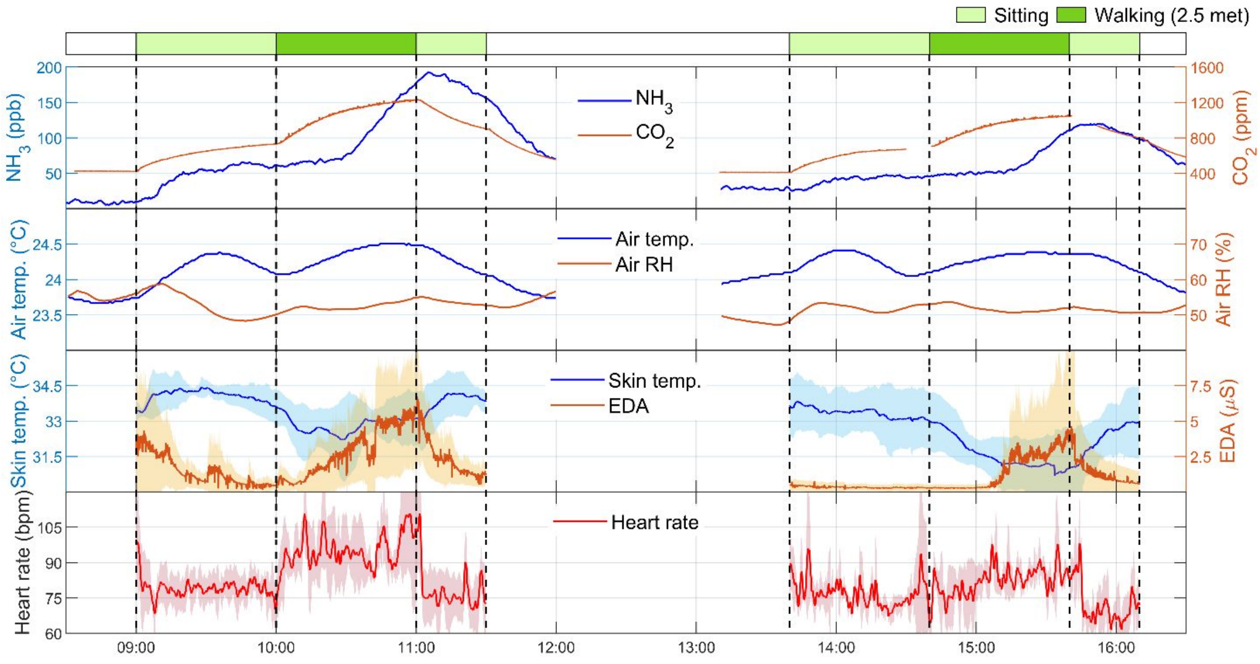

Fig. S2.

Time-series of NH_3 and CO_2 concentration, chamber air temperature and relative humidity, and physiological data from participants: skin temperature and EDA, and heart rate in the experiments of physiological engagement by walking at 2.5 met. Date: 19/08/2022. The morning and afternoon experiments were performed with participant groups G2A and G2B, respectively. The lines of the physiological data represent averages of all the three participants in each group. Shaded areas represent standard deviation.

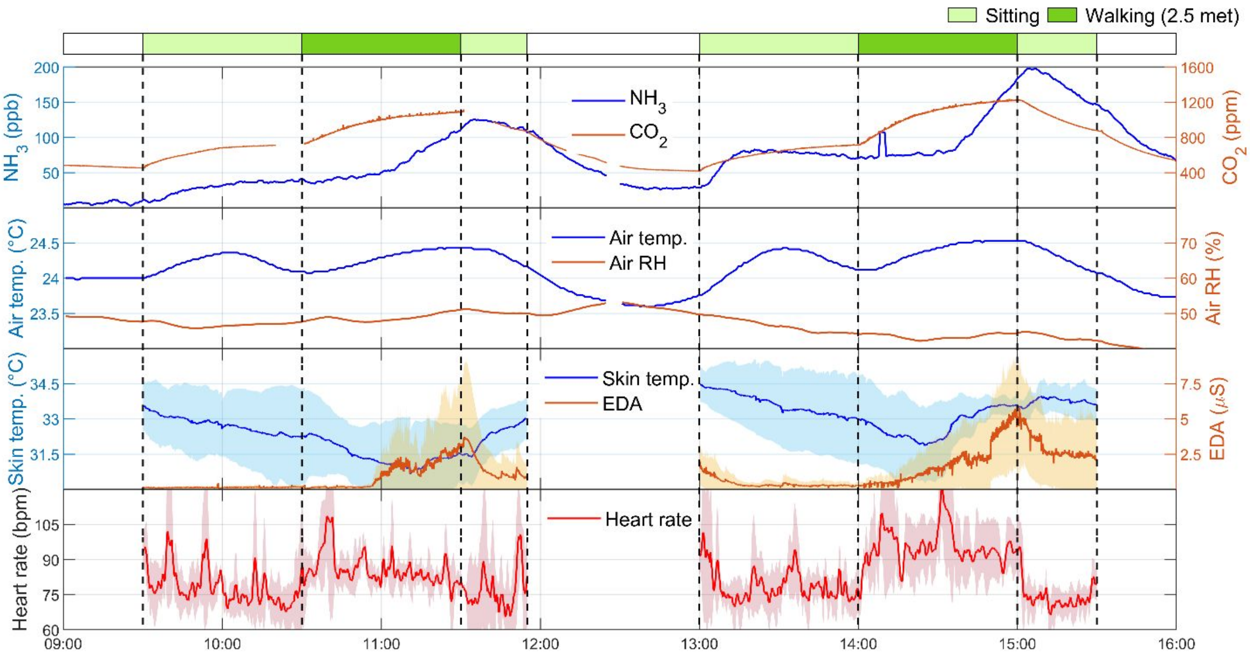

Fig. S3.

Time-series of NH_3 and CO_2 concentration, chamber air temperature and relative humidity, and physiological data from participants: skin temperature and EDA, and heart rate in the experiments of physiological engagement by walking at 2.5 met. Date: 22/08/2022. The morning and afternoon experiments were performed with participant groups G2B and G2A, respectively. The lines of the physiological data represent averages of all the three participants in each group. Shaded areas represent standard deviation.

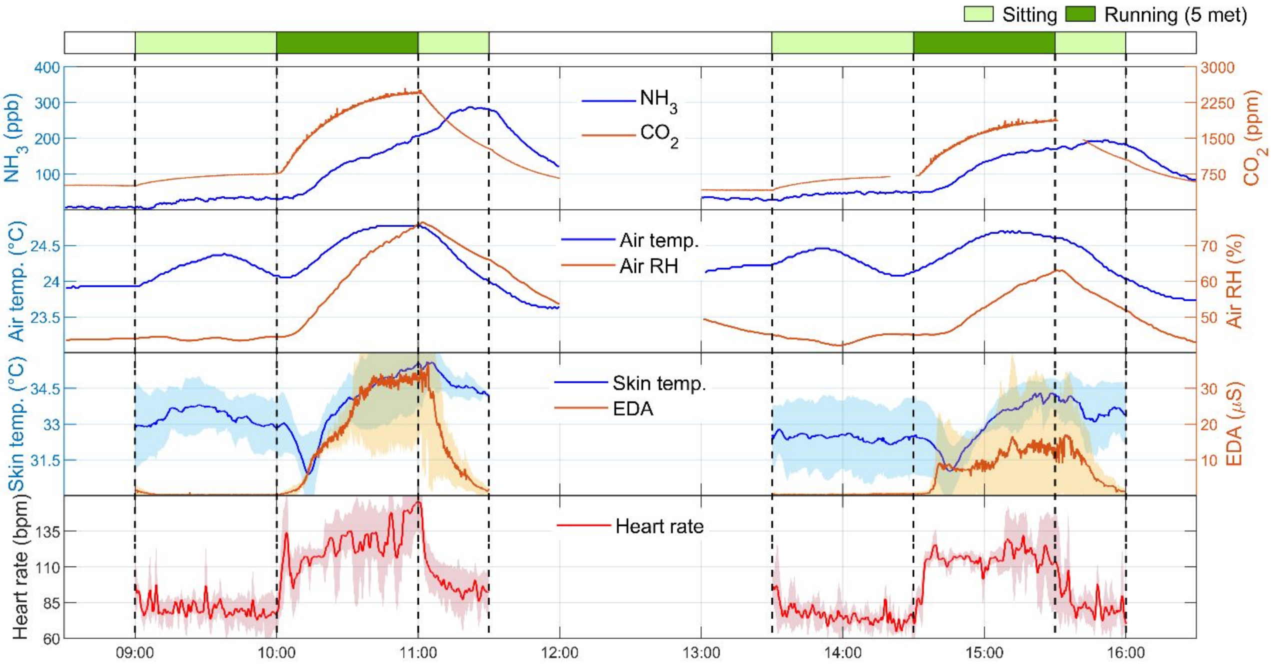

Fig. S4.

Time-series of NH_3 and CO_2 concentration, chamber air temperature and relative humidity, and physiological data from participants: skin temperature and EDA, and heart rate in the experiments of physiological engagement by running at 5 met. Date: 23/08/2022. The morning and afternoon experiments were performed with participant groups G2A and G2B, respectively. The lines of the physiological data represent averages of all the three participants in each group. Shaded areas represent standard deviation.

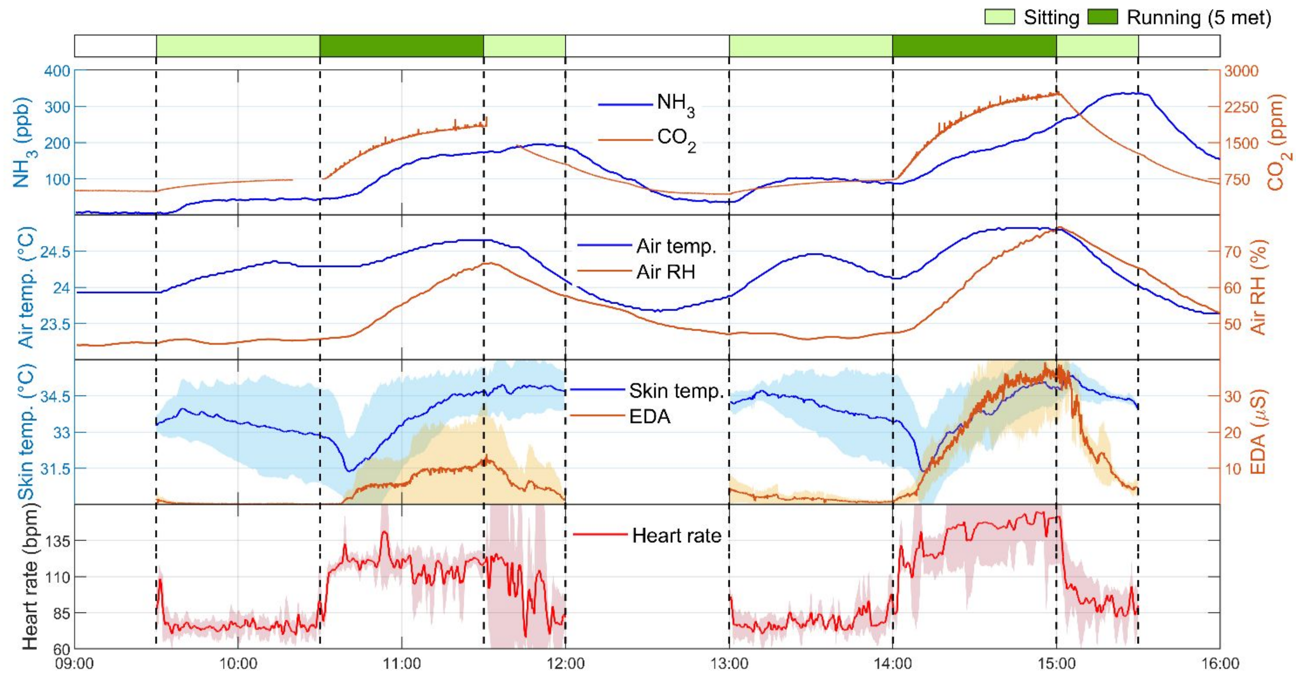

Fig. S5.

Time-series of NH_3 and CO_2 concentration, chamber air temperature and relative humidity, and physiological data from participants: skin temperature and EDA, and heart rate in the experiments of physiological engagement by running at 5 met. Date: 24/08/2022. The morning and afternoon experiments were performed with participant groups G2B and G2A, respectively. The lines of the physiological data represent averages of all the three participants in each group. Shaded areas represent standard deviation.

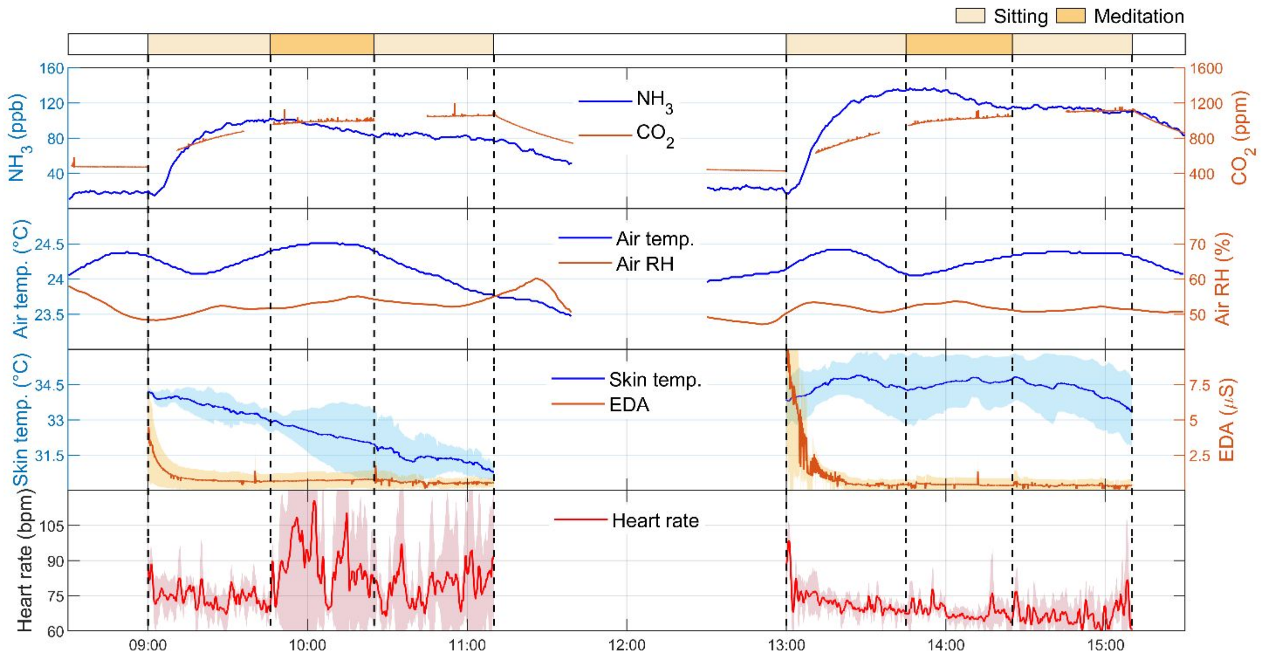

Fig. S6.

Time-series of NH_3 and CO_2 concentration, chamber air temperature and relative humidity, and physiological data from participants: skin temperature and EDA, and heart rate in the experiments of psychological engagement by meditation. Date: 26/08/2022. The morning and afternoon experiments were performed with participant groups G3A and G3B, respectively. The lines of the physiological data represent averages of all the three participants in each group. Shaded areas represent standard deviation.

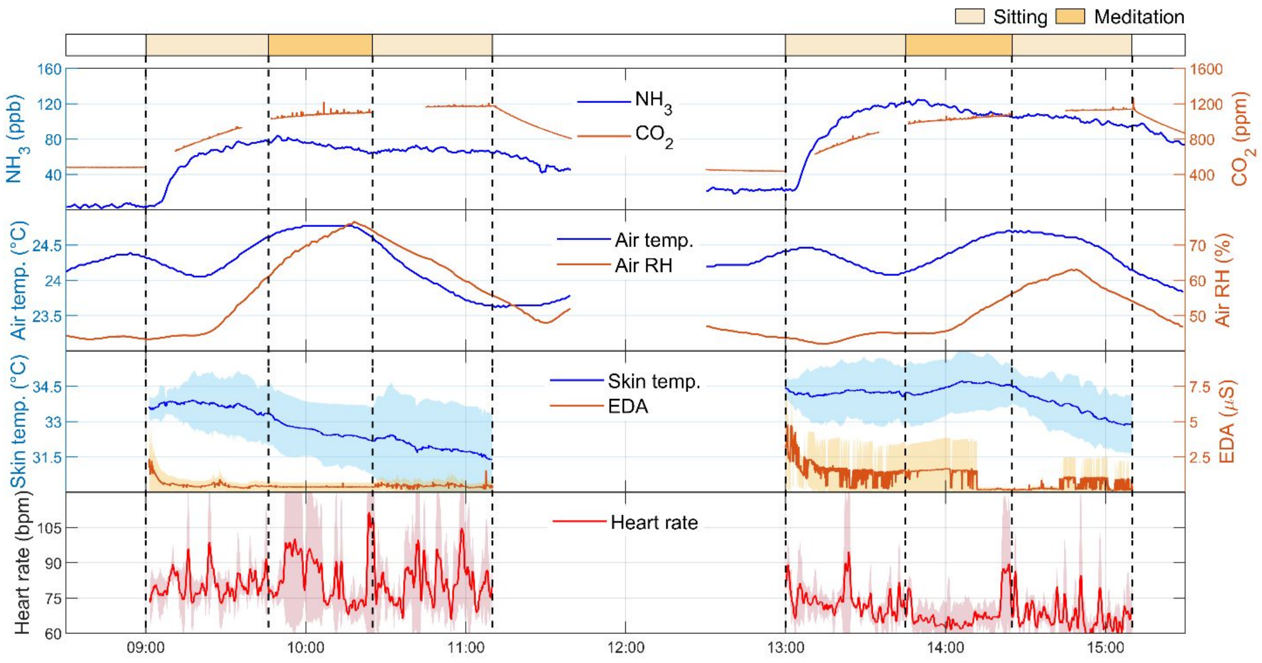

Fig. S7.

Time-series of NH_3 and CO_2 concentration, chamber air temperature and relative humidity, and physiological data from participants: skin temperature and EDA, and heart rate in the experiments of psychological engagement by meditation. Date: 30/08/2022. The morning and afternoon experiments were performed with participant groups G3A and G3B, respectively. The lines of the physiological data represent averages of all the three participants in each group. Shaded areas represent standard deviation.

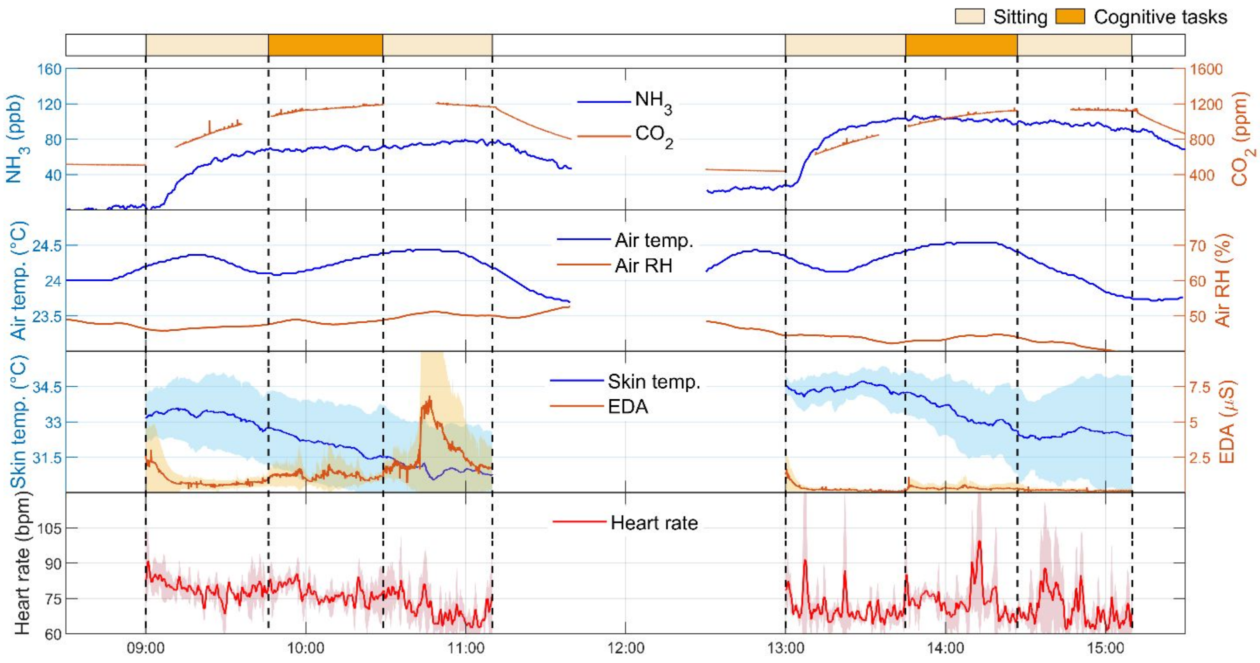

Fig. S8.

Time-series of NH_3 and CO_2 concentration, chamber air temperature and relative humidity, and physiological data from participants: skin temperature and EDA, and heart rate in the experiments of psychological engagement by cognitive tasks. Date: 29/08/2022. The morning and afternoon experiments were performed with participant groups G3A and G3B, respectively. The lines of the physiological data represent averages of all the three participants in each group. Shaded areas represent standard deviation. Note that the peak of EDA in the morning from G3A was due to one participant briefly leaving the chamber for the restroom.

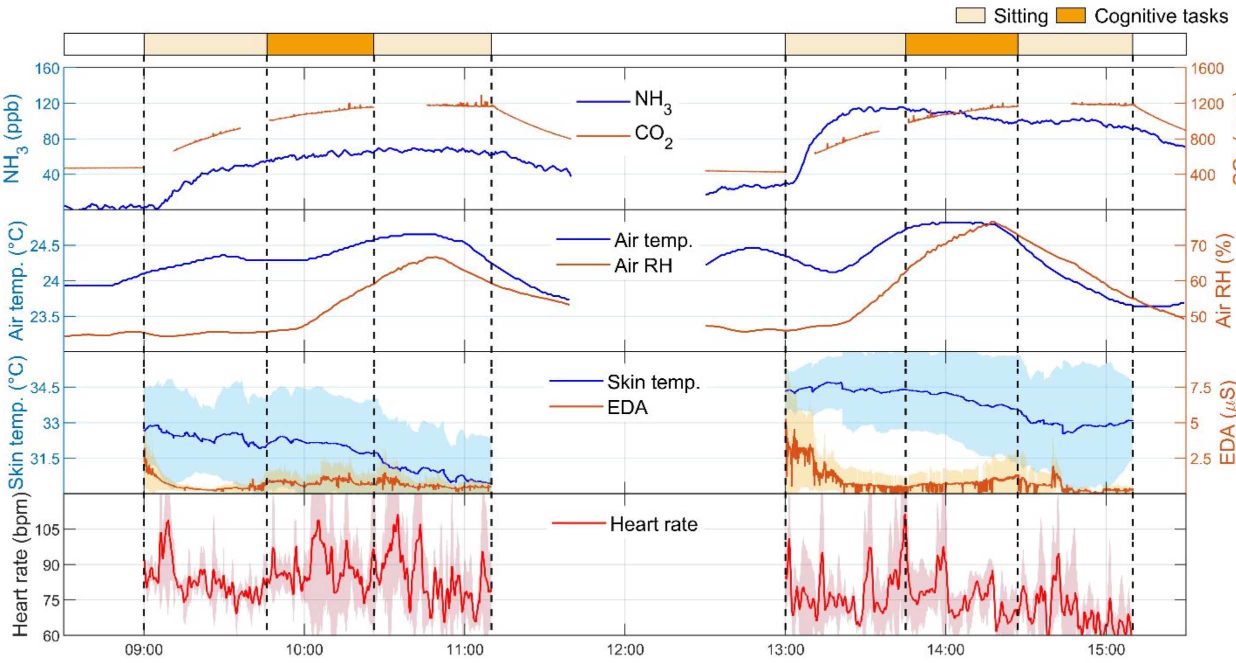

Fig. S9.

Time-series of NH_3 and CO_2 concentration, chamber air temperature and relative humidity, and physiological data from participants: skin temperature and EDA, and heart rate in the experiments of psychological engagement by cognitive tasks. Date: 31/08/2022. The morning and afternoon experiments were performed with participant groups G3A and G3B, respectively. The lines of the physiological data represent averages of all the three participants in each group. Shaded areas represent standard deviation.

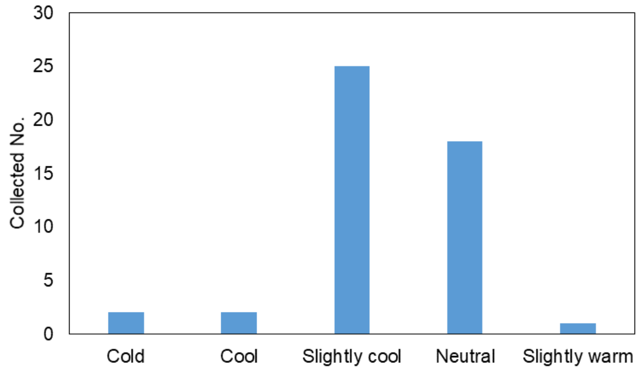

Fig. S10.

Thermal perception of participants collected 30 min after entering the chamber across all the experiments.

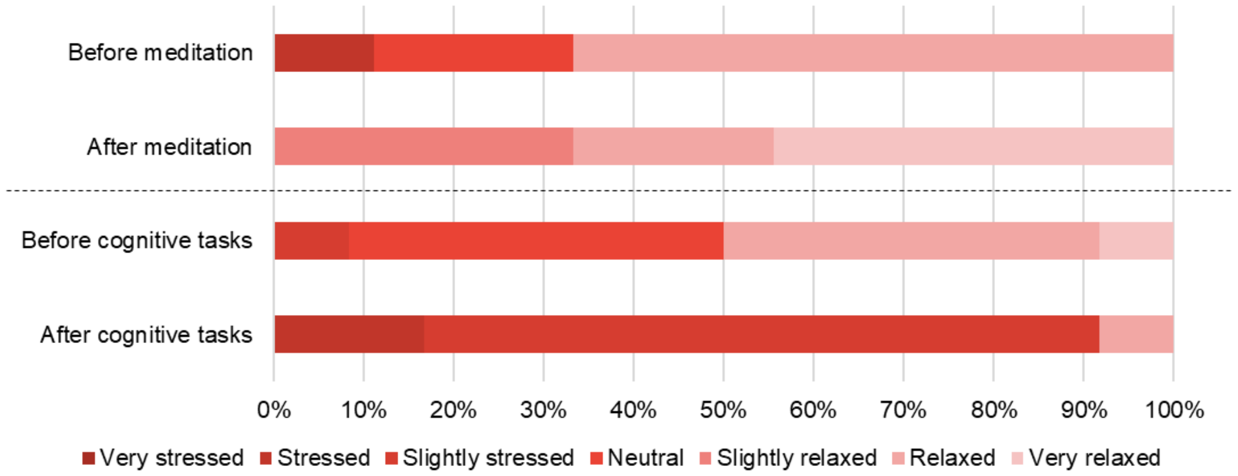

Fig. S11.
Self-reported stress level from participants immediately before and after the 40-min psychological engagement session.

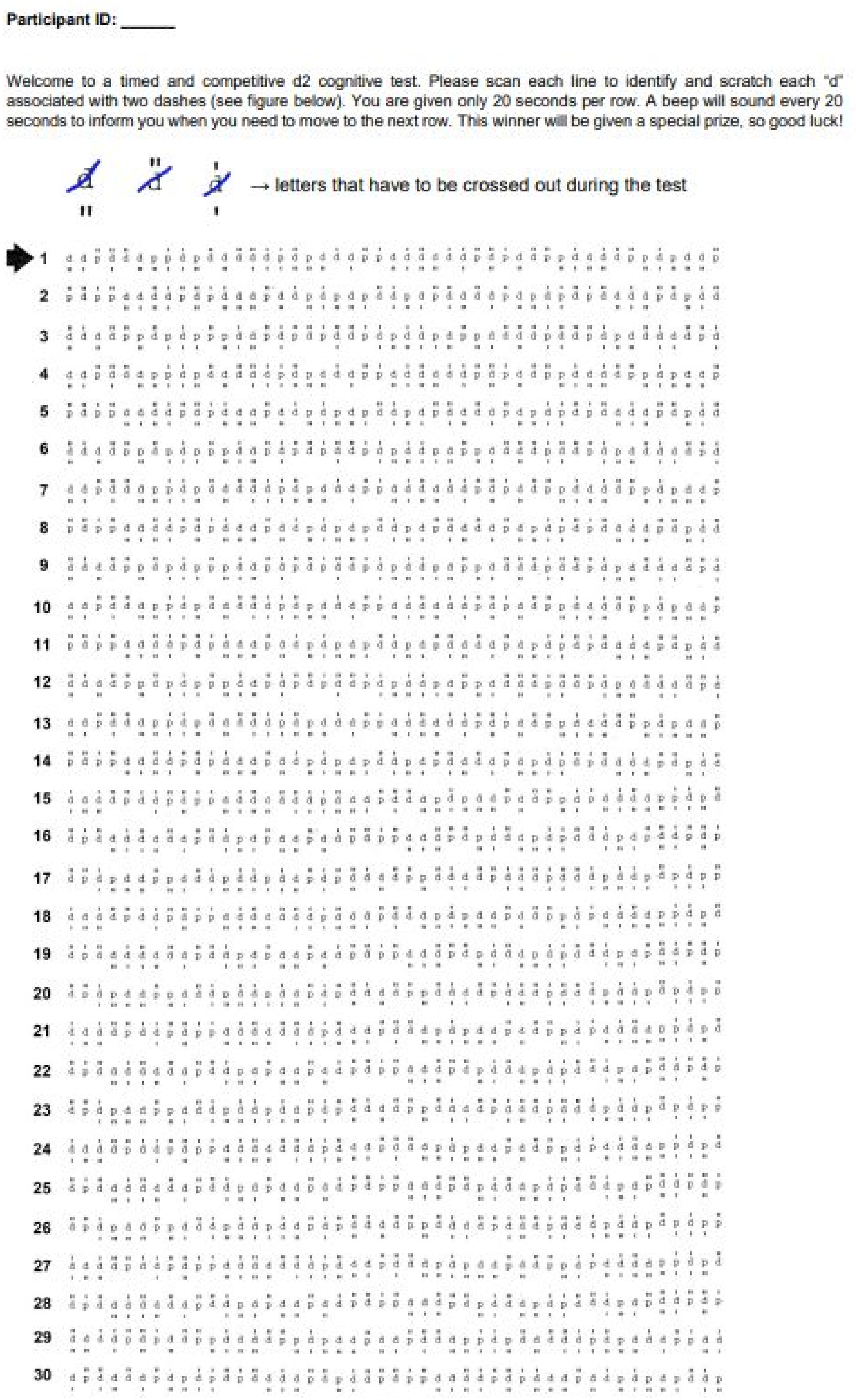

Participant ID: ______

Welcome to a timed and competitive d2 cognitive test. Please scan each line to identify and scratch each "d" associated with two dashes (see figure below). You are given only 20 seconds per row. A beep will sound every 20 seconds to inform you when you need to move to the next row. This winner will be given a special prize, so good luck!

→ letters that have to be crossed out during the test

Fig. S12.
d2 Test sheet used in the psychological engagement experiments.

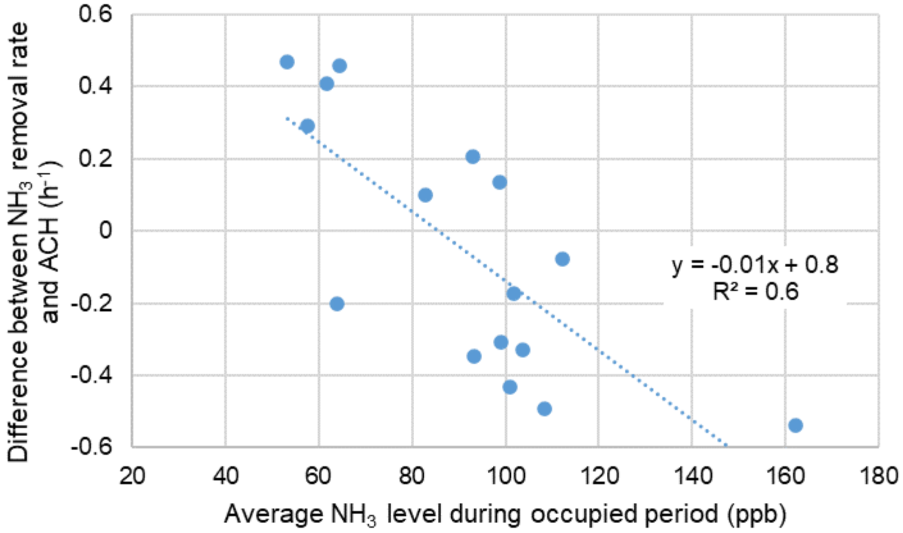

Fig. S13.
The difference between NH_3 removal rate and air change rate (ACH) in relation to average NH_3 level during occupied period in each experiment.

Table S1.
Physiological data of participants in each group and treadmill settings for each participant in the physiological engagement experiments (inc. is the inclination index set on the treadmill)

						Walking (2.5 met)		Running (5 met)	
Group	Gender	Age	Height (cm)	Weigh t (kg)	BMI (kg/m^2)	Nominal heart rate (bpm)	Treadmill setting	Nominal heart rate (bpm)	Treadmill setting
G2A	Male	29	181	96	29.3	87	3 km/h, 0 inc.	113	5.5 km/h, 15 inc.
	Female	24	168	70	24.8	94	2.8 km/h, 0 inc.	133	5.5 km/h, 15 inc.
	Male	24	180	83	25.6	88	3.5 km/h, 0 inc.	117	5.5 km/h, 15 inc.
G2B	Female	24	174	65	21.5	94	2.5 km/h, 0 inc.	132	5 km/h, 15 inc.
	Male	19	180	70	21.6	89	3 km/h, 0 inc.	120	6 km/h, 12 inc.
	Female	32	165	59	21.7	95	3 km/h, 0 inc.	139	5.5 km/h, 15 inc.
G3A	Male	29	181	96	29.3	--	--	--	--
	Female	32	165	59	21.7	--	--	--	--
	Male	24	180	83	25.6	--	--	--	--
G3B	Male	24	187	84	24.0	--	--	--	--
	Female	30	165	60	22.0	--	--	--	--
	Male	19	180	70	21.6	--	--	--	--

Table S2.

Summary of experimental conditions and associated average CO_2 and NH_3 emission rates, human physiological data, and chamber temperature and humidity. CO_2 emission rate (L/h per person), NH_3 emission rate (mg/h per person), Skin T: skin temperature (°C), EDA: electrodermal activity (μS), HR: Heart rate (bpm), Air T: chamber air temperature (°C), Air RH: chamber air relative humidity (%)

					Before engagement							During engagement							After engagement						
Date 2022	AM /PM	Engage	Session	Group	CO_2	NH_3	Skin T	ED -A	HR	Air T	Air RH	CO_2	NH_3	Skin T	ED-A	HR	Air T	Air RH	CO_2	NH_3	Skin T	ED-A	HR	Air T	Air RH
0819	AM	Physio.	Walking 2.5 met	G2A	19.6	3.0	34.1	1.5	79.2	24.2	52.8	51.9	6.2	32.8	2.8	94.9	24.3	52.3	23.1	7.5	33.8	2.4	78.3	24.3	53.7
0819	PM	Physio.	Walking 2.5 met	G2B	17.8	2.2	33.4	0.3	76.9	24.2	51.9	40.5	3.9	31.6	1.5	82.1	24.3	51.8	17.8	4.8	32.2	1.3	72.1	24.3	51.2
0822	AM	Physio.	Walking 2.5 met (Rep.)	G2B	20.2	1.8	32.8	0.1	78.5	24.2	46.8	43.0	3.9	31.5	1.0	85.8	24.3	48.7	20.2	5.4	32.3	1.6	76.9	24.3	50.4
0822	PM	Physio.	Walking 2.5 met (Rep.)	G2A	18.9	3.9	33.7	0.4	79.1	24.2	46.6	52.4	6.2	32.8	1.8	95.3	24.4	43.5	21.5	6.9	33.7	3.0	74.2	24.3	43.4
0823	AM	Physio.	Running 5 met	G2A	21.6	1.5	33.3	0.3	80.7	24.2	43.9	135.5	8.2	33.7	19.6	124.5	24.5	60.5	30.1	14.1	34.8	13.6	101.6	24.4	71.2
0823	PM	Physio.	Running 5 met	G2B	18.4	2.3	32.4	0.3	76.2	24.3	43.9	96.1	7.3	32.9	9.1	114.6	24.5	52.8	20.4	8.8	33.6	7.7	84.7	24.3	57.3
0824	AM	Physio.	Running 5 met (Rep.)	G2B	20.5	2.2	33.4	0.2	76.6	24.2	45.2	93.0	7.5	33.2	6.3	116.6	24.5	55.3	21.5	9.1	34.8	6.3	101.2	24.4	62.3
0824	PM	Physio.	Running 5 met (Rep.)	G2A	19.6	4.8	34.1	1.6	78.9	24.3	46.6	137.0	10.2	33.6	22.1	138.0	24.6	62.4	28.7	16.6	34.7	16.8	97.2	24.4	70.9
0826	AM	Psycho.	Meditation	G3A	17.0	2.6	33.6	1.0	74.4	24.2	50.6	14.2	1.3	32.4	0.7	88.5	24.5	53.3	15.3	1.3	31.4	0.6	79.2	24.1	53.1
0826	PM	Psycho.	Meditation	G3B	17.4	3.5	34.5	1.6	72.5	24.3	51.9	16.1	1.8	34.6	0.4	68.1	24.2	52.6	16.8	1.8	34.3	0.4	66.7	24.4	51.3
0829	AM	Psycho.	Cognitive tasks	G3A	20.5	1.8	33.3	0.8	79.0	24.3	46.6	19.6	1.2	32.1	1.2	77.3	24.2	48.2	16.1	1.4	31.0	2.9	70.6	24.4	50.2
0829	PM	Psycho.	Cognitive tasks	G3B	17.0	2.6	34.4	0.2	70.6	24.2	43.8	19.2	1.7	33.4	0.3	74.3	24.5	43.8	16.0	1.5	32.5	0.2	70.4	24.1	41.6
0830	AM	Psycho.	Meditation (Rep.)	G3A	20.0	2.1	33.7	0.5	80.8	24.2	48.3	17.1	1.0	32.6	0.4	83.8	24.7	70.8	17.8	1.2	31.9	0.5	81.9	24.0	65.1
0830	PM	Psycho.	Meditation (Rep.)	G3B	18.4	3.1	34.2	1.6	73.3	24.3	43.7	16.5	1.6	34.5	1.1	67.8	24.4	48.6	17.2	1.6	33.6	0.5	67.2	24.5	59.2
0831	AM	Psycho.	Cognitive tasks (Rep.)	G3A	19.0	1.4	32.4	0.4	82.7	24.3	45.1	19.7	1.2	32.1	0.8	88.0	24.4	50.9	17.1	1.1	30.9	0.6	84.1	24.6	63.4
0831	PM	Psycho.	Cognitive tasks (Rep.)	G3B	18.8	2.9	34.4	1.4	78.1	24.3	50.8	20.2	1.6	34.0	0.7	76.5	24.8	71.5	17.3	1.5	32.9	0.4	69.6	24.0	64.1

Table S3.

Multilinear regression coefficients for CO_2 and NH_3 emission rates with human physiological data (skin temperature, EDA, and heart rate) and air temperature and relative humidity. The regression used average data across each sub-session in all experiments (48 data points in total). $^{**}p<0.01$, $^{***}p<0.001$

	**Skin temperature**	**EDA**	**Heart rate**	**Air temperature**	**Air relative humidity**	**Intercept**	**Adj. R^2**
CO_2	-1.80<br>[p=0.4]	1.55<br>[p=0.04]	1.32 ***<br>[p=10^{-6}]	14.68<br>[p=0.3]	-0.82<br>[p=0.4]	-339.23	0.81
NH_3	0.68 **<br>[p=0.01]	0.49 ***<br>[p=0.0001]	0.01<br>[p=0.7]	-2.17<br>[p=0.3]	0.03<br>[p=0.6]	30.26	0.67

Table S4.

NH_3 removal rate in each experiment after participants left the chamber and comparison with air change rate (ACH). Ave: average; SD: standard deviation

Date 2022	AM /PM	Engage	Session	Group	NH_3 removal rate (h^{-1})	NH_3 removal rate (h^{-1}) Ave ± SD	ACH (h^{-1}) Ave ± SD	Difference
0819	AM	Physio.	Walking 2.5 met	G2A	2.52	2.58 ± 0.24	2.87 ± 0.04	10.1%
0819	PM	Physio.	Walking 2.5 met	G2B	2.67			
0822	AM	Physio.	Walking 2.5 met (Rep.)	G2B	3.16			
0822	PM	Physio.	Walking 2.5 met (Rep.)	G2A	2.54			
0823	AM	Physio.	Running 5 met	G2A	2.38			
0823	PM	Physio.	Running 5 met	G2B	2.44			
0824	AM	Physio.	Running 5 met (Rep.)	G2B	2.56			
0824	PM	Physio.	Running 5 met (Rep.)	G2A	2.33			
0826	AM	Psycho.	Meditation	G3A	1.54	1.63 ± 0.23	1.44 ± 0.01	13.2%
0826	PM	Psycho.	Meditation	G3B	1.36			
0829	AM	Psycho.	Cognitive tasks	G3A	1.85			
0829	PM	Psycho.	Cognitive tasks	G3B	1.65			
0830	AM	Psycho.	Meditation (Rep.)	G3A	1.90			
0830	PM	Psycho.	Meditation (Rep.)	G3B	1.27			
0831	AM	Psycho.	Cognitive tasks (Rep.)	G3A	1.91			
0831	PM	Psycho.	Cognitive tasks (Rep.)	G3B	1.57			
